# Identification of CFH and FHL2 as biomarkers for idiopathic pulmonary fibrosis

**DOI:** 10.3389/fmed.2024.1363643

**Published:** 2024-05-09

**Authors:** Xingchen Liu, Meng Yang, Jiayu Li, Hangxu Liu, Yuchao Dong, Jianming Zheng, Yi Huang

**Affiliations:** ^1^Department of Pathology, The First Affiliated Hospital of Naval Medical University, Navy Medical University, Shanghai, China; ^2^Department of Respiratory and Critical Care Medicine, The First Affiliated Hospital of Naval Medical University, Navy Medical University, Shanghai, China; ^3^Department of Breast and Thyroid Surgery, The First Affiliated Hospital of Naval Medical University, Navy Medical University, Shanghai, China

**Keywords:** CFH, FHL2, biomarker, IPF, machine-learning strategies

## Abstract

**Background:**

Idiopathic pulmonary fibrosis (IPF) is a fatal disease of unknown etiology with a poor prognosis, characterized by a lack of effective diagnostic and therapeutic interventions. The role of immunity in the pathogenesis of IPF is significant, yet remains inadequately understood. This study aimed to identify potential key genes in IPF and their relationship with immune cells by integrated bioinformatics analysis and verify by *in vivo* and *in vitro* experiments.

**Methods:**

Gene microarray data were obtained from the Gene Expression Omnibus (GEO) for differential expression analysis. The differentially expressed genes (DEGs) were identified and subjected to functional enrichment analysis. By utilizing a combination of three machine learning algorithms, specific genes associated with idiopathic pulmonary fibrosis (IPF) were pinpointed. Then their diagnostic significance and potential co-regulators were elucidated. We further analyzed the correlation between key genes and immune infiltrating cells via single-sample gene set enrichment analysis (ssGSEA). Subsequently, a single-cell RNA sequencing data (scRNA-seq) was used to explore which cell types expressed key genes in IPF samples. Finally, a series of *in vivo* and *in vitro* experiments were conducted to validate the expression of candidate genes by western blot (WB), quantitative real-time PCR (qRT-PCR), and immunohistochemistry (IHC) analysis.

**Results:**

A total of 647 DEGs of IPF were identified based on two datasets, including 225 downregulated genes and 422 upregulated genes. They are closely related to biological functions such as cell migration, structural organization, immune cell chemotaxis, and extracellular matrix. CFH and FHL2 were identified as key genes with diagnostic accuracy for IPF by three machine learning algorithms. Analysis using ssGSEA revealed a significant association of both CFH and FHL2 with diverse immune cells, such as B cells and NK cells. Further scRNA-seq analysis indicated CFH and FHL2 were specifically upregulated in human IPF tissues, which was confirmed by *in vitro* and *in vivo* experiments.

**Conclusion:**

In this study, CFH and FHL2 have been identified as novel potential biomarkers for IPF, with potential diagnostic utility in future clinical applications. Subsequent investigations into the functions of these genes in IPF and their interactions with immune cells may enhance comprehension of the disease’s pathogenesis and facilitate the identification of therapeutic targets.

## 1 Introduction

IPF is a chronic progressive lung disease distinguished by the excessive accumulation of mesenchymal cells and extracellular matrix, resulting in an irreversible decline in lung function (1). Predominantly affecting individuals in middle to older age groups, the incidence rate of IPF is steadily rising by 11% annually (2). Due to the insidious onset of IPF, the difficulty of imaging and histology to distinguish IPF from other usual interstitial lung diseases (ILDs), and the absence of reliable laboratory tests contribute to delayed diagnoses in most patients, typically occurring during the intermediate to advanced disease stages (1, 3). Because of delayed diagnosis and limited treatment options, individuals with IPF experience a median survival time of 2 to 5 years, with a five-year survival rate of 30%, which is inferior to that of many malignancies (4, 5).

At present, the pathogenesis of IPF is not fully understood, and it is mainly related to repetitive damage and repair dysregulation of alveolar epithelial cells. Epithelial apoptosis and impaired function of progenitors (AT2 cells) lead to the inability to complete the normal re-epithelialization process, induce fibroblasts to proliferate and transform into myofibroblasts. These activated myofibroblasts then deposit extracellular matrix (ECM) components, such as collagen, and exert contractile forces to facilitate wound healing (6). Scar foci formation triggers restrictive ventilation disorders and gas exchange disorders, ultimately leading to respiratory failure and death (7). Studies have found that immunity and inflammation are closely related to IPF, in which both innate and adaptive immunity are activated (8). In IPF, the damaged pulmonary epithelial cells released chemokines and cytokines, which leads to the recruitment and activation of innate immune cells such as neutrophils and macrophages, further activate the adaptive immune system (B cells and T cells) (9). Single-cell analysis showed that increased alveolar macrophages, dendritic cells (DCs), and memory T cells were present in IPF lungs and had possessed an activation profile indicating increased IFN-γ signaling and upregulation of adaptive immunity (10). Multicellular interactions between the activated innate and adaptive immune cells and lung fibroblasts may be crucial for the pathologic mechanisms of IPF and need further, which required further research.

Currently, there is a lack of effective treatments for IPF. Only two recommended drugs (nintedanib and pirfenidone) that have been approved for IPF but can only delay the decline in lung function and cannot stop the progression of the disease (11, 12). Numerous clinical trials investigating the efficacy of anti-inflammatory drugs for IPF have yielded unfavorable outcomes, including potential harm to patients (13, 14).

In summary, IPF remains a fatal disease characterized by a lack of timely diagnosis and efficacious treatment modalities. Therefore, this article aims to find the biomarkers with significantly altered expression levels in IPF patients through bioinformatics analysis, evaluate their diagnostic efficacy, and explore the relationship between the key genes that may be found and immune cell infiltration, so as to offer novel perspectives for the development of targeted immunotherapies for IPF.

## 2 Materials and methods

### 2.1 Dataset collection

**Figure 1:**
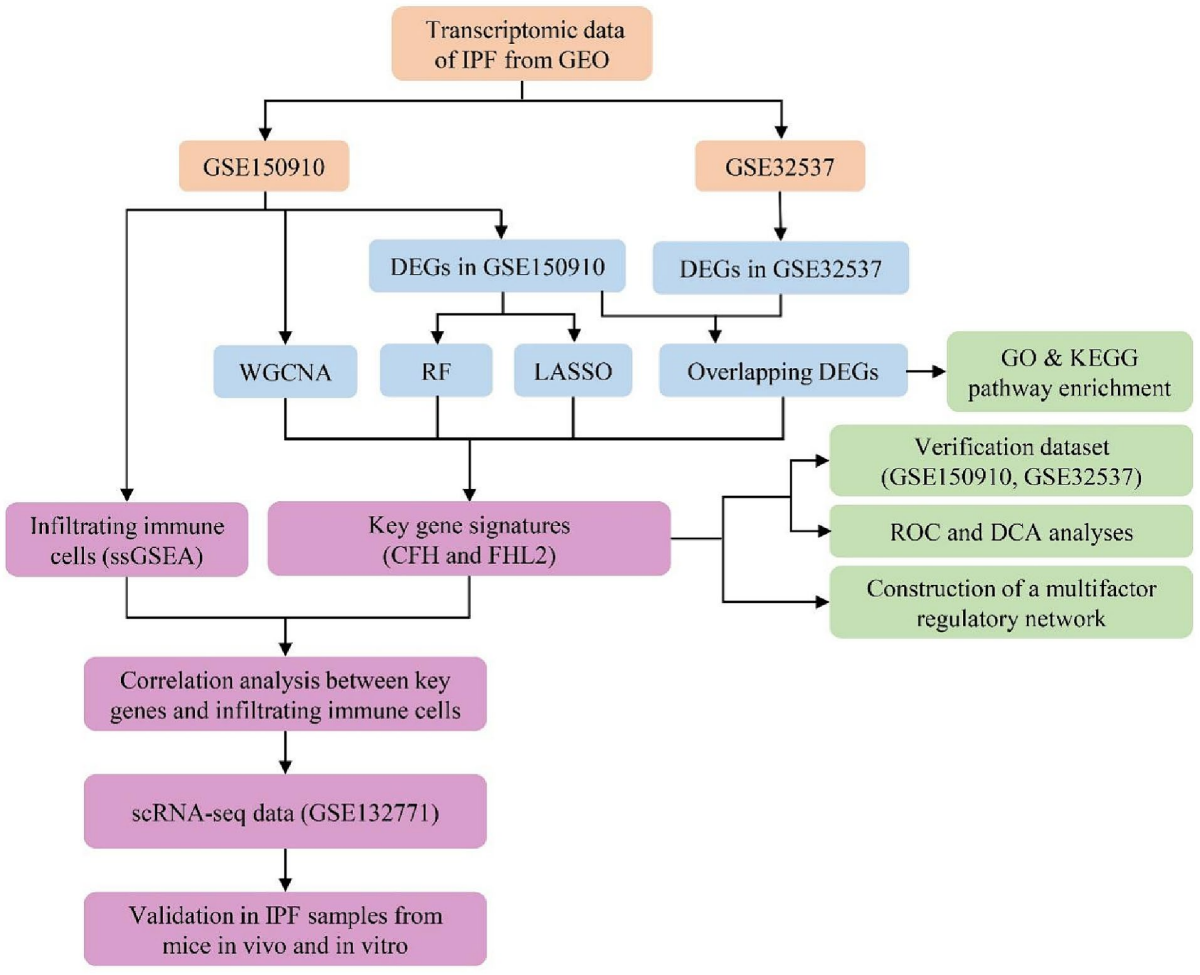
Flowchart of the study.

Data analysis procedures of our study are shown in Figure 1. Gene expression profiles of GSE150910 and GSE32537 were downloaded from the Gene Expression Omnibus (GEO) database. GSE150910 dataset includes RNA-sequencing results of 103 IPF lung samples and 103 unaffected control lung samples. GSE32537 dataset includes transcriptional profiles on lung tissue from 119 IPF subjects and 50 non-diseased controls. The scRNA-seq data, accession number GSE132771, was obtained from GEO based on GPL24676 platform. We used sequencing results of three IPF patient lungs and three normal human lungs.

### 2.2 Identification of DEGs

We conducted the differential expression analysis by comparing IPF lung samples to normal lung samples in the R computing environment using limma package. Genes were regarded as differentially expressed with the threshold of FDR-adjusted *p*-value <0.05 and |Log2foldchange (FC)| ≥ 0.585. FDRs were estimated with Benjamini-Hochberg procedure. Visualization of DEGs including volcano plots, heatmaps for top 50 DEGs, and Venn diagram was achieved by using ggplot2 package, Pheatmap package, and VennDiagram package in R, respectively.

### 2.3 Functional enrichment analysis

After identifying the overlapping DEGs between the above two datasets, we performed Gene Ontology (GO) and Kyoto Encyclopedia of Genes and Genomes (KEGG) analysis via the clusterProfiler package to determine function and pathway enrichment of DEGs. Enrichment is achieved by comparing GO terms or pathways present in DEGs using three annotation databases of GO terms (BP, Biological Process; CC, Cellular Component; and MF, Molecular Function) or KEGG pathway database. The GO enrichment was performed for all DEGs together and for up- and down-regulated genes separately. Only terms with *p*-values and q-values<0.05 were considered significantly enriched. The results were visualized with GOplot R package.

### 2.4 Machine learning algorithms

In order to reduce the risk of bias, three machine learning algorithms were applied in this study to select characteristic genes of IPF: RF, Random Forest, LASSO logistic regression, The Least Absolute Shrinkage and Selection Operator, and WGCNA, Weighted Gene Co-expression Network Analysis. All statistical analyses were implemented through the randomForest package, glmnet package, and WGCNA package of R software, respectively. The results of the three algorithms were intersected by Venn diagrams to finalize the possible key genes for further study.

### 2.5 Diagnostic efficacy assessment

The expression levels of key genes identified were compared between the IPF group and the control group in two gene databases separately. If the differential expression is significant (*p*-value <0.05 and |Log2FC| ≥ 0.585), the diagnostic effectiveness of key genes was evaluated by plotting the ROC curve and calculating the AUC via R software pROC package. These genes were considered to be poorly diagnostic if the AUC is less than 0.7, moderately diagnostic if 0.7–0.9, and well diagnostic if greater than 0.9. In addition, we compared the predicted clinical assessment effects of key genes by constructing decision curve analysis (DCA).

### 2.6 Mapping gene regulatory network

Gene expression is subject to multiple regulators. We analyzed microRNAs (miRNA), long non-coding RNA (lncRNA), and transcription factor (TF) associated with the expression of key genes through mirDIP, starbase, and hTFtarget to find regulators that may lead to differential expression. The visualization process and the search for common regulators between key genes were completed by Cytoscape software.

### 2.7 Immune cell infiltration

First, we analyzed the intra-group similarity and inter-group variability between the IPF group and the control group by principal component analysis (PCA) to evaluate the sample data quality. ssGSEA was used to calculate the abundance of immune cell infiltrated in each sample from GSE150910 and plot heat map. The differences in Infiltrated immune cell set and function between the IPF and the control group were compared by ssGSEA and box plots were drawn. Spearman rank correlation coefficient calculation was used to screen infiltrating immune cells closely related to key genes. *p* < 0.05 was considered statistically significant.

### 2.8 Single-cell RNA sequencing analysis

The barcodes data, gene features data, and gene count matrix data of GSE132771 preprocessed by Cellranger (10X Genomics) were downloaded from the GEO database. We conducted the differential expression analysis by comparing IPF lung samples to normal lung samples in the R computing environment using the Seurat V4.1.0 package. Firstly, cells were subjected to quality control based on the following criteria: a gene count per cell >500, a percentage of mitochondrial genes <20%, and a red blood cell gene proportion < 3%. Then the data were normalized by SCTransform function (15) and integrated with reciprocal principal component analysis (rPCA) approach (16). Subsequently, PCA, cluster analysis, and Uniform Manifold Approximation and Projection (UMAP) were performed using the RunPCA, FindClusters, and RunUMAP functions, respectively. Additionally, cell annotation was performed using the SingleR V1.4.1 package with the HumanPrimaryCellAtlasData as the reference dataset (17).

### 2.9 Isolation, culture and treatment of primary lung fibroblasts

Lung tissue from euthanized post-natal 4 weeks SD rats were quickly extracted (trachea and excess tissue removed) and rinsed with PBS before being infiltrated in serum-free medium. Then we minced lung tissue into 1–3 mm pieces with sterile ophthalmic forceps in a dish and transferred pieces to serum-free medium containing Dispase II (Sigma-Aldrich, 4,942,078,001), DNase I (Sigma-Aldrich, D5025). After incubating at 37°C for 45 min with gentle shaking every 15 min, add 10% FBS to stop digestion. Digestion products passing through a 70 μm filter were centrifuged at 1500 rpm for 5 min. Added Red Cell Lysis Buffer (Beyotime, C3702) to resuspend, incubated at room temperature for 5 min, and then centrifuged at 1500 rpm for 5 min. After resuspending cells with DMEM with 10% FBS (Gibco, United States), 100 U/mL of penicillin and 100 μg/mL of streptomycin, PLFs were plated at a seeding density of 5 × 10^5^–1 × 10^6^/well with 2 mL complete medium in 6-well plate. All steps were performed on ice or at 4°C unless stated otherwise. Cells were cultured at 37°C in a 5% CO2 incubator with regular feeds and passaged at 80% fusion using 0.25% of trypsin–EDTA in a 1:3 ratio. PLFs were divided into three groups comprising a control group of cells without treatment, a group with TGFβ1 10 ng/mL treatment for 48 h, and a group with TGFβ1 10 ng/mL treatment for 72 h.

### 2.10 Bleomycin-induced mouse lung fibrosis model

10 wild-type SPF-grade male C57BL/6 J mice (6 weeks) were purchased from Shanghai Bikai Keyi Biotechnology Co. and randomly divided into two groups, a control group (*n* = 5) and a bleomycin group (*n* = 5). After gas anesthesia, the mice were intratracheally injected with normal saline (total volume 50 μL) or 5 mg/kg Bleomycin (selleck, S1214). Lung tissue samples from the mice were collected at 21 days.

### 2.11 Hematoxylin and eosin, Masson staining and immunohistochemistry

H&E staining and Masson staining was performed as previously described (18). Briefly, lung specimens were fixed with 4% paraformaldehyde for 24 h. Then the samples were dehydrated, paraffin embedded and cut into 3 μm sections. Sections were stained with hematoxylin and eosin (H&E) and Masson’s trichrome stain to assess gross morphology and collagen deposition, respectively. For IHC, after dewaxing and hydration, epitope retrieval was performed with 10 mM citrate buffer. Then sections were blocked with 1% BSA for 1 h at ambient temperature before incubated with primary antibody at 4°C overnight. Then the sections were rewarmed for 45 min on the next day and incubated with secondary antibodies for 30 min at room temperature (Zsbio, pv8000), followed by detection using the DAB detection kit (OriGene Technologies, ZLI-9017). The primary antibodies and secondary antibodies used were as follows: anti-CFH (Abclonal, A13686; 1:100), anti-FHL (proteintech, 21,619-1-AP; 1:100) and anti-α-SMA (Abcam, ab7817; 1:100).

### 2.12 Quantitative real-time PCR

Total RNA was extracted using the RNA extraction Kit (Fastagen, 220,010). Reverse transcription was performed using cDNA Synthesis Kit (Vazyme, R312-01). The reverse transcription conditions were 37°C for 15 min and 85°C for 5 s. The RT-PCR were performed using HiScript RT superMix for qPCR (Vazyme, R122-01) and the reaction conditions were initial denaturation at 95°C for 10 min, followed by 40 cycles of 95°C for 15 s and 60°C for 45 s. Primers and reagents used were as follows: Fhl2(rat), Forward: 5′- TCTGACCCCACAGGTTGCTG-3′; Reverse: 5′- TCACAGGTGTTGGCATAGAGC-3′. Cfh(rat), Forward: 5′- GTGTAAAGCCCCGAAGTCAAC-3′; Reverse: 5′- GGAGGGCAGAATCTTTTCTCATT-3′. Acta2 (rat), Forward: 5′- GTGTTCAGAGAGGGTGAGCC-3′; Reverse: 5′- TCAGGTTGGTCCTCTGGTCT-3′. Gapdh (rat), Forward: 5′- GCATCTTCTTGTGCAGTGCC-3′; Reverse: 5′- GATGGTGATGGGTTTCCCGT-3′.

### 2.13 Western blot

Samples were lysed with RIPA buffer on ice and centrifuged at 16000 g for 15 min to extract protein. Protein concentrations were measured using the BCA Protein Assay kit (Thermo, 23,227). These protein samples were separated by electrophoresis using 8% or 15% SDS-PAGE at 100 V for 20 min and 120 V for 100 min. Proteins were electrostatically transferred to NC membrane and blocked with 5% BSA for 120 min. The primary antibodies were incubated overnight at 4°C and the secondary antibody for 60 min at room temperature. Finally, the labeled protein bands were developed with developing solution and scanned. All experiments were repeated three times. The antibodies used were as follows: GADPH (Abcam, 16,891; 1:1000), anti-CFH (Abclonal, A13686; 1:1000), anti-FHL (proteintech, 21,619-1-AP; 1:1000) and anti-α-SMA (Abcam, ab7817; 1:1000).

### 2.14 Statistical analyses

All statistical analyses were performed using GraphPad Prism 8.0. A two-tailed unpaired student *t*-test was used to determine significance. One-way analysis of variance (ANOVA) with a Bonferroni post-test was used to compare differences among multiple groups. *p* < 0.05 was considered as statistically significant.

## 3 Results

### 3.1 Identification of DEGs

**Figure 2:**
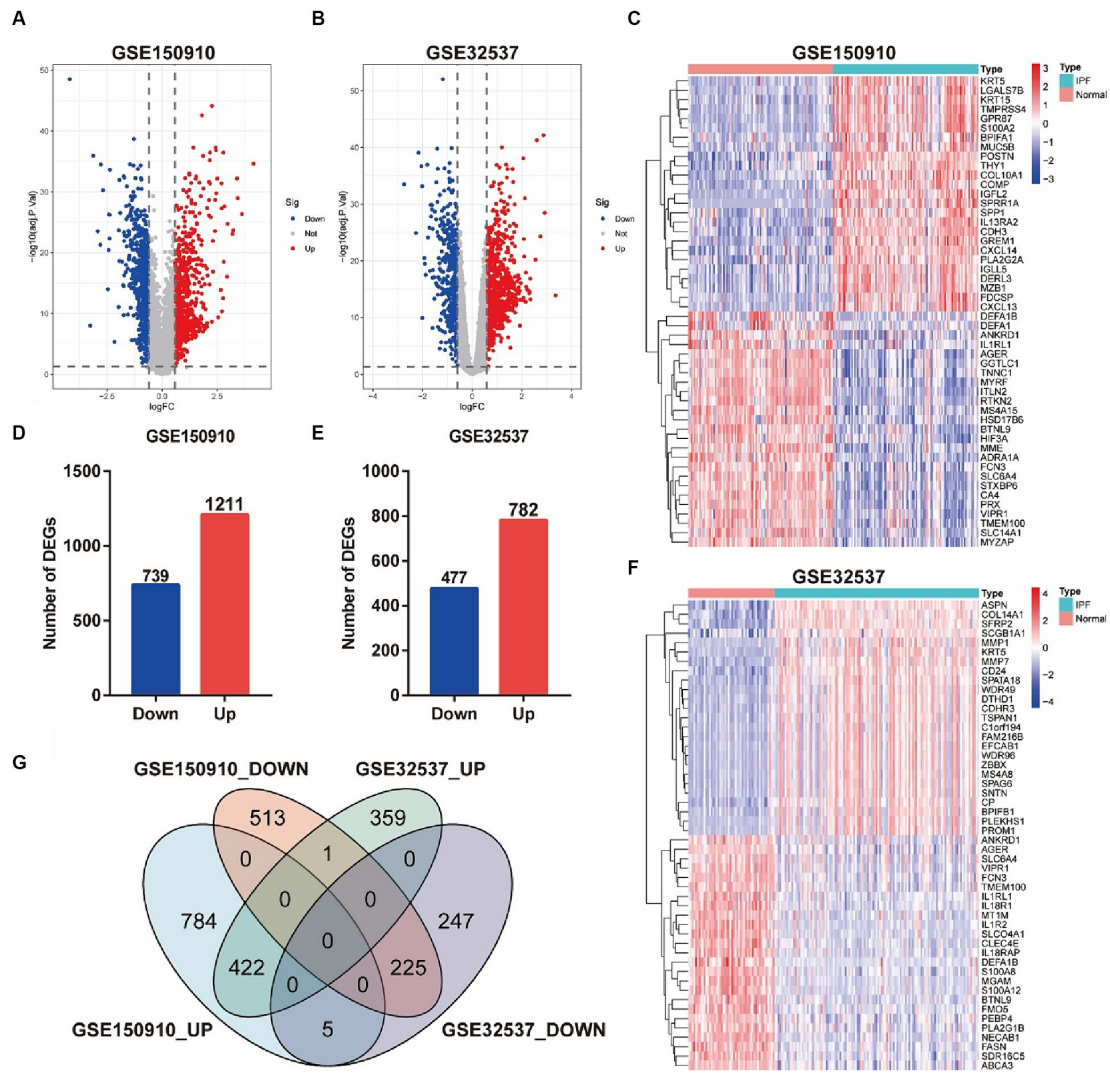
Identification of DEGs between IPF and normal samples. **(A,B)** Volcano plots of genes in GSE150910 **(A)** and GSE32537 **(B)**. Red dots represent up-regulated genes, blue dots represent down-regulated genes, and gray dots represent genes with no significance (|log2FC| > 0.585 and FDR < 0.05). **(C,F)** Heatmaps of top 25 up- and down-regulated DEGs in GSE150910 **(C)** and GSE32537 **(F)**. x-axis represents each sample, and y-axis represents each gene. Legend on the top right represents the log fold change of the genes. Red and blue colors represent relative increase or decrease in gene expression. **(D,E)** Number of DEGs in GSE150910 **(D)** and GSE32537 **(E)**. **(G)** Venn diagram of DEGs from the two datasets.

Differential expression analysis showed that there were 1950 differentially expressed genes (DEGs) in the GSE150910, including 739 genes down-regulated in the IPF group and 1,211 genes up-regulated (Figures 2A,D). Meanwhile, there were 1,259 DEGs in the GSE32537, consisted of 477 down-regulated genes and 782 up-regulated genes (Figures 2B,E). The comparison of gene expression between the IPF group and the control group is shown by the volcano plot (Figures 2A,B). The top 25 up-regulated genes and the top 25 down-regulated genes in the GSE150910 or GSE32537 were presented in the heat maps, respectively, (Figures 2C,F). The Venn diagrams exhibited a total of 225 overlapping down-regulated genes and 422 overlapping up-regulated genes between the two datasets (Figure 2G).

### 3.2 Functional enrichment analysis

**Figure 3:**
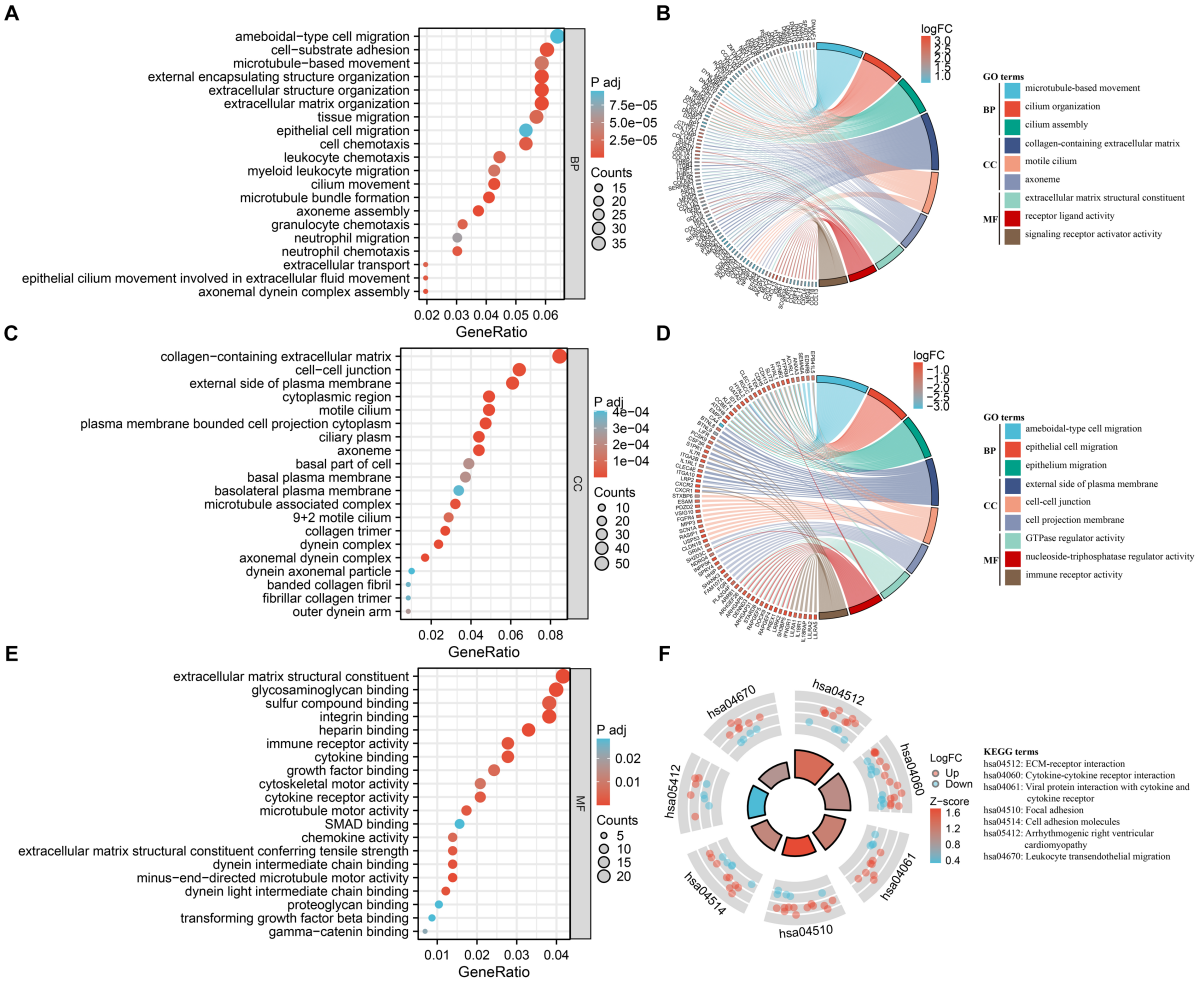
Functional enrichment analysis of DEGs. **(A,C,E)** GO analysis of DEGs. Bubble charts indicate enriched GO terms associated with DEGs in IPF sorted by: BP **(A)**, CC **(C)**, and MF **(E)**. x-axis represents gene ratios, and y-axis represents GO terms. Circle size represents gene count and color represents adjusted *p*-value. **(B,D)** Chord plots demonstrate enriched GO terms of up-regulated DEGs **(B)** and down-regulated DEGs **(D)**. The colored squares next to each gene indicate the logFC values shown in the legend on the top right. Each DEG is connected to their respective GO terms by ribbons, and the color of the GO term corresponds to the ribbon. **(F)** KEGG pathway enrichment analysis of DEGs. The outside ring shows the expression levels (logFC) of each gene in KEGG pathway. Red dots represent up-regulated DEGs, and blue dots represent down-regulated DEGs. The inside ring is a bar plot, with the height indicating the significance of the pathway enrichment and color indicating z-score shown in the legend on the right.

A biological functional classification of overlapping DEGs was performed by GO enrichment analysis. In the BP category, the significantly enriched terms included ameboidal-type cell migration, cell-substrate adhesion, microtubule-based movement, external encapsulating structure organization, extracellular structure organization, extracellular matrix organization, tissue migration, epithelial cell migration, and cell chemotaxis (Figure 3A). Meanwhile the significantly enriched terms in CC contained collagen-containing extracellular matrix, cell–cell junction, and external side of plasma membrane (Figure 3C). As for MF, the significantly enriched terms comprised extracellular matrix structural constituent, glycosaminoglycan binding, sulfur compound binding, integrin binding, and heparin binding (Figure 3E). Subsequently, GO enrichment analysis were conducted on the up-regulated and down-regulated overlapping DEGs, respectively (Figures 3B,D). Among the up-regulated DEGs, the predominantly enriched go terms included microtubule-based movement, cilium organization, and cilium assembly in BP, collagen-containing extracellular matrix, motile cilium, and axoneme in CC, and extracellular matrix structural constituent, receptor ligand activity, and signaling receptor activator activity in MF. In the down-regulated DEGs, the remarkably enriched go terms included ameboidal-type cell migration, epithelial cell migration, and epithelium migration in BP, external side of plasma membrane, cell–cell junction, and cell projection membrane in CC, and GTPase regulator activity, nucleoside-triphosphatase regulator activity, and immune receptor activity in MF. Then pathway analyses were performed by mapping genes to KEGG pathways. The result showed that the most abundant pathways included ECM-receptor interaction, cytokine-cytokine receptor interaction, viral protein interaction with cytokine and cytokine receptor, and focal adhesion (Figure 3F).

### 3.3 Feature selection

**Figure 4:**
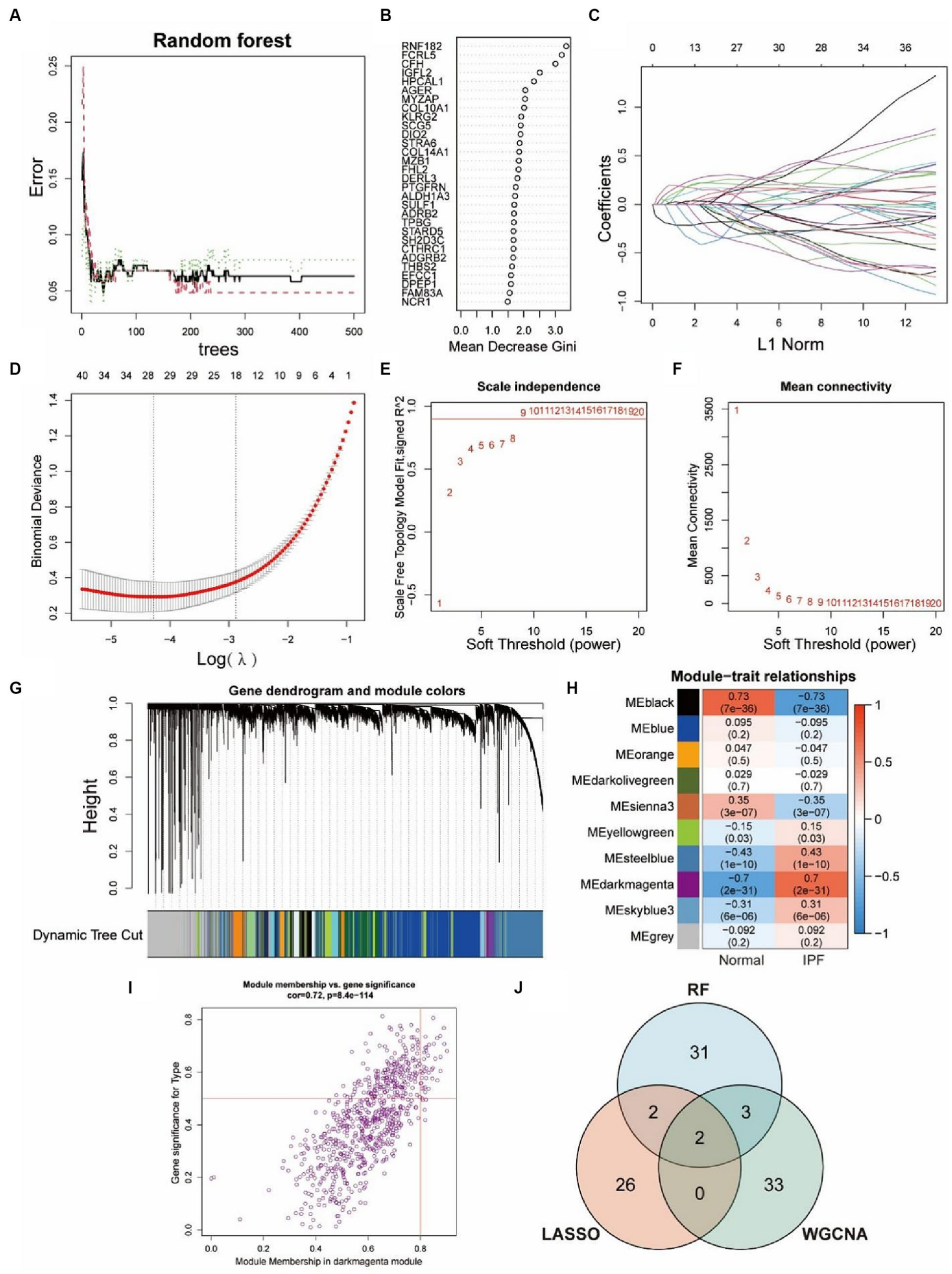
Identification of key genes via three machine-learning algorithms. **(A,B)** Key genes selection via RF algorithm. Distribution of out-of-bag (OOB) error rate at various values of trees **(A)**. Variable importance assessed in terms of the mean decrease Gini is computed using the OOB error **(B)**. A higher mean decrease in Gini coefficient indicates higher variable importance. **(C,D)** Key genes selection via LASSO algorithm. LASSO coefficient profile of 29 genes, different colors represent different genes **(C)**. Selection of the optimal parameter (lambda) in the LASSO model, and generation of a coefficient profile plot **(D)**. **(E–I)** Key genes selection via WGCNA algorithm. Network topology analysis of various soft-threshold powers **(E,F)**. Horizontal axis represents soft threshold power, and vertical axis represents scale free topology model fit index **(E)** or mean connectivity **(F)**. Clustering dendrogram of DEGs related to IPF **(G)**, with dissimilarity based on topological overlap, together with assigned module colors. Module-trait associations **(H)**. Each row corresponds to a module, and each column corresponds to a trait. Each cell contains corresponding correlation and *p*-value. The table is color-coded by correlation according to the color legend. Gene significance for IPF in the MEdarkmagenta module **(I)**. One dot represents one gene in the module. Venn diagram shows the intersection of key genes obtained by three indicated algorithms **(J)**.

Three machine learning algorithms were combined to analyze the data from GSE150910 and screen for key genes by taking their intersections. 38 genes were identified by RF algorithm (Figures 4A,B) and 30 genes were screened out by LASSO regression algorithm (Figures 4C,D). In the WGCNA analysis, the network was constructed with 9 as the soft threshold based on the scale-free topology model fit index and the mean connectivity (Figures 4E,F). We identified 10 modules that were significantly co-expressed (Figure 4G) and explored the correlation between each module and IPF through a heat map (Figure 4H). The result showed that the MEdarkmagenta module had the highest positive correlation with IPF, so we further screened 38 genes that were highly correlated with IPF from the MEdarkmagenta module (Figure 4I). The Venn diagram showed that CFH and FHL2 were the overlapping key genes screened by three algorithms ultimately (Figure 4J).

### 3.4 Diagnostic value of key genes

**Figure 5:**
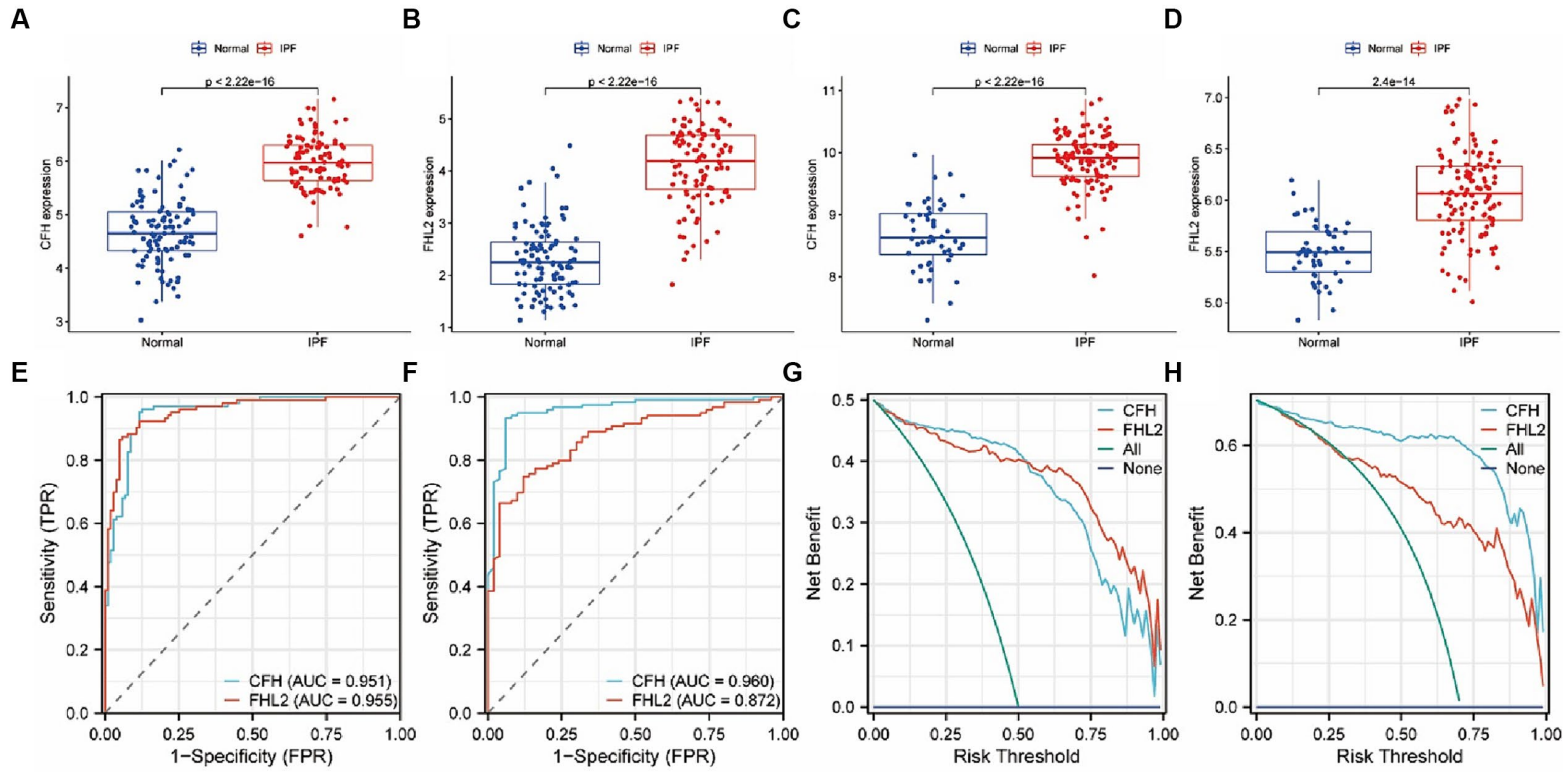
the expression levels of CFH and FHL2 in two datasets. **(A–D)** Box plots of the expression of CFH and FHL2 between IPF and normal samples in GSE150910 **(A,B)** and GSE32537 **(C,D)**. **(E,F)** The ROC curves of CFH and FHL2 in GSE150910 **(E)** and GSE32537 **(F)**. **(G,H)** The decision curves of CFH and FHL2 in GSE150910 **(G)** and GSE32537 **(H)**. CFH, complement factor H; FHL2, four and a half LIM domains 2.

By comparing the expression levels of CFH and FHL2 between the IPF group and the control group in the GSE150910 and GSE32537 datasets, the significant high expression of the two key genes in the disease group was verified (*p* < 0.01) (Figures 5A–D). The ROC curves indicated that both CFH and FHL2 exhibited strong diagnostic capabilities, with CFH achieving an AUC of 0.951 or 0.960, and FHL2 an AUC of 0.955 or 0.872 (Figures 5E,F). In the DCA curves, the net benefit of CFH method or FHL2 method was higher than that of the two extreme curves (all treatment or no treatment) within a large risk threshold range, which meant the two diagnostic methods had good clinical utility (Figures 5G,H).

### 3.5 Gene regulatory network

**Figure 6:**
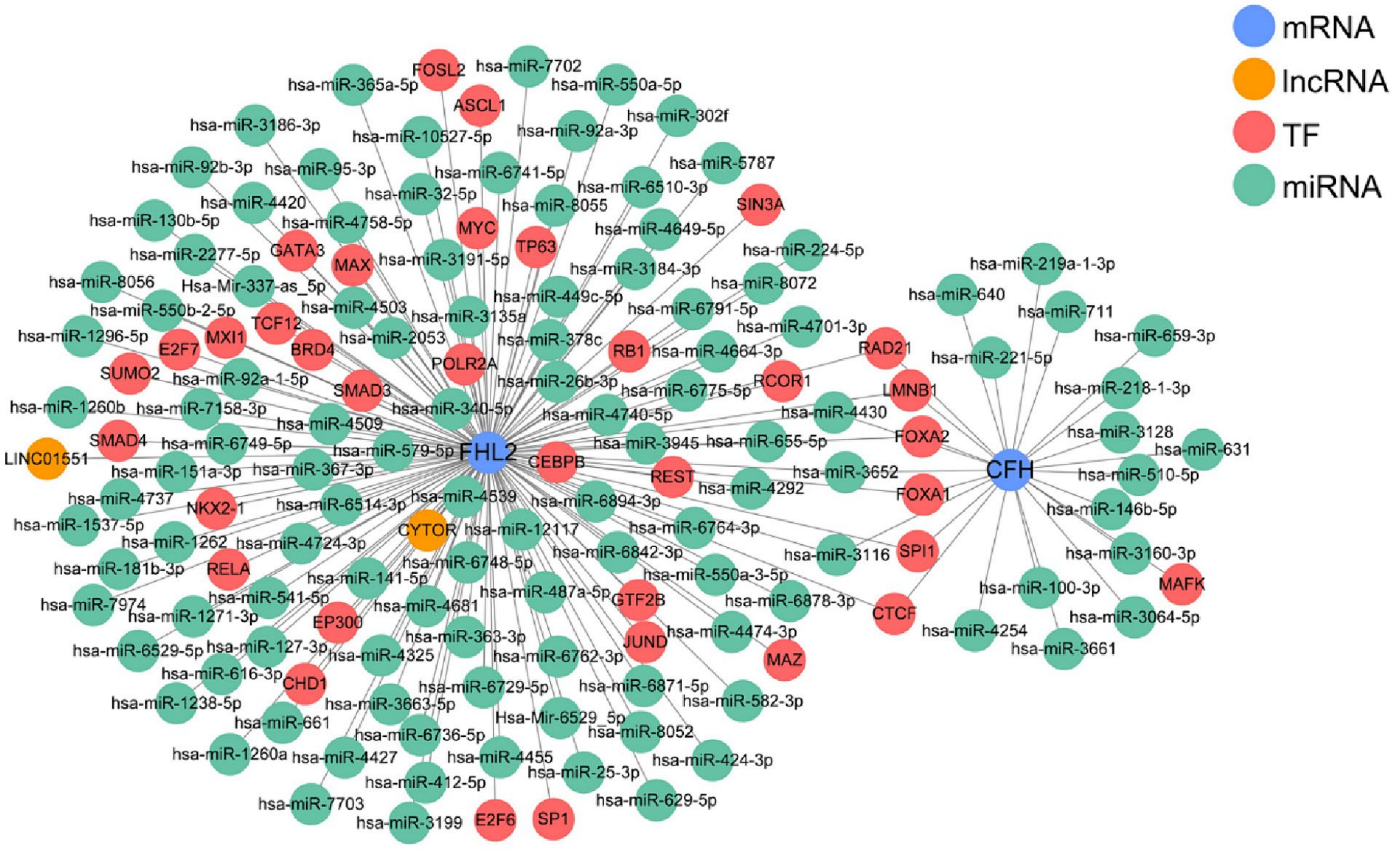
The multifactor regulatory network based on CFH and FHL2.

By integrating lncRNA/miRNA/TF that interacts with two key genes, we constructed a gene multifactor regulatory network. In this network, CFH was regulated by a total of 18 miRNAs and 7 TFs, while FHL2 was regulated by 98 miRNAs, 2 lncRNAs and 34 TFs. Notably, the regulatory network revealed the presence of TFs that co-regulate two key genes, including RAD21, LMNB1, FOXA2, FOXA1, SPI1, and CTCF (Figure 6).

### 3.6 Assessment of immune cell infiltration

**Figure 7:**
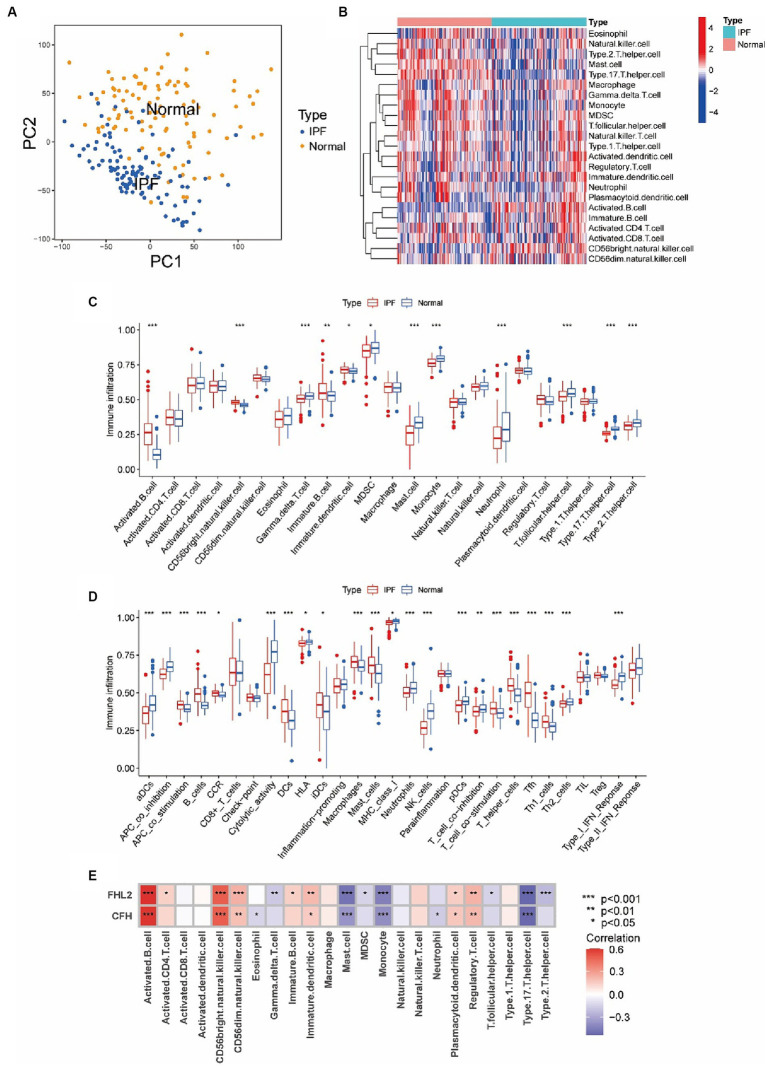
Assessment of immune cell infiltration and its relationship to key genes in IPF. **(A)** Principal component analysis (PCA) cluster plot of gene expression profile between IPF and normal samples in GSE150910. **(B)** Heatmap of infiltrating immune cells in GSE150910. x-axis represents each sample, and y-axis represents each cell types. Legend on the top right represents the log fold change of cell counts. Red and blue colors represent high and low cell counts. **(C,D)** Box plots of the proportion of 23 types of immune cells **(C)** and 29 types of immune functions **(D)** between IPF and normal samples. *p < 0.05, **p < 0.01, ***p < 0.001. **(E)** Correlations between CFH, FHL2, and infiltrating immune cells. Each cell is color-coded for logFC value of the correlation according to legend on the right, and significance is indicated.

The existing literature suggested that immunity is closely related to IPF, so we further explored the relationship between immune cell infiltration and two key genes. First, we confirmed by PCA that the samples in the IPF group and the control group from the GSE150910 dataset were well separated (Figure 7A). Then heatmap of infiltrating immune cells in GSE150910 was achieved (Figure 7B). Next, we compared the composition of 23 types of infiltrated immune cells between the IPF group and the control group. Box plot displayed that the IPF group had a higher proportion of activated B cell, immature B cell, and CD56bright NK cell (all *p* < 0.01), and a lower proportion of mast cell, monocyte, neutrophil, γδT cell, Tfh, Th17, and Th2 (all *p* < 0.001) (Figure 7C). By comparing the immune function score, it was found that APC co-stimulation, DC, macrophage, mast cell, B cell, Th, Tfh, Th1, and T cell co-stimulation was increased and aDC, pDC, neutrophil, NK cell, APC co-inhibition, cytolytic activity, Th2, and type1 IFN response was decreased in the IPF group (all *p* < 0.001) (Figure 7D). The correlation analysis between genes and immune cells demonstrated that both FHL2 and CFH were positively correlated with activated B cell, CD56bright NK cell, CD56dim NK cell and Treg, and negatively correlated with mast cell, monocyte, and Th17. Furthermore, FHL2 displayed a positive correlation with immature DC and a negative correlation with γδT cell and Th2 (all *p* < 0.01) (Figure 7E).

### 3.7 Single cell gene expression

**Figure 8:**
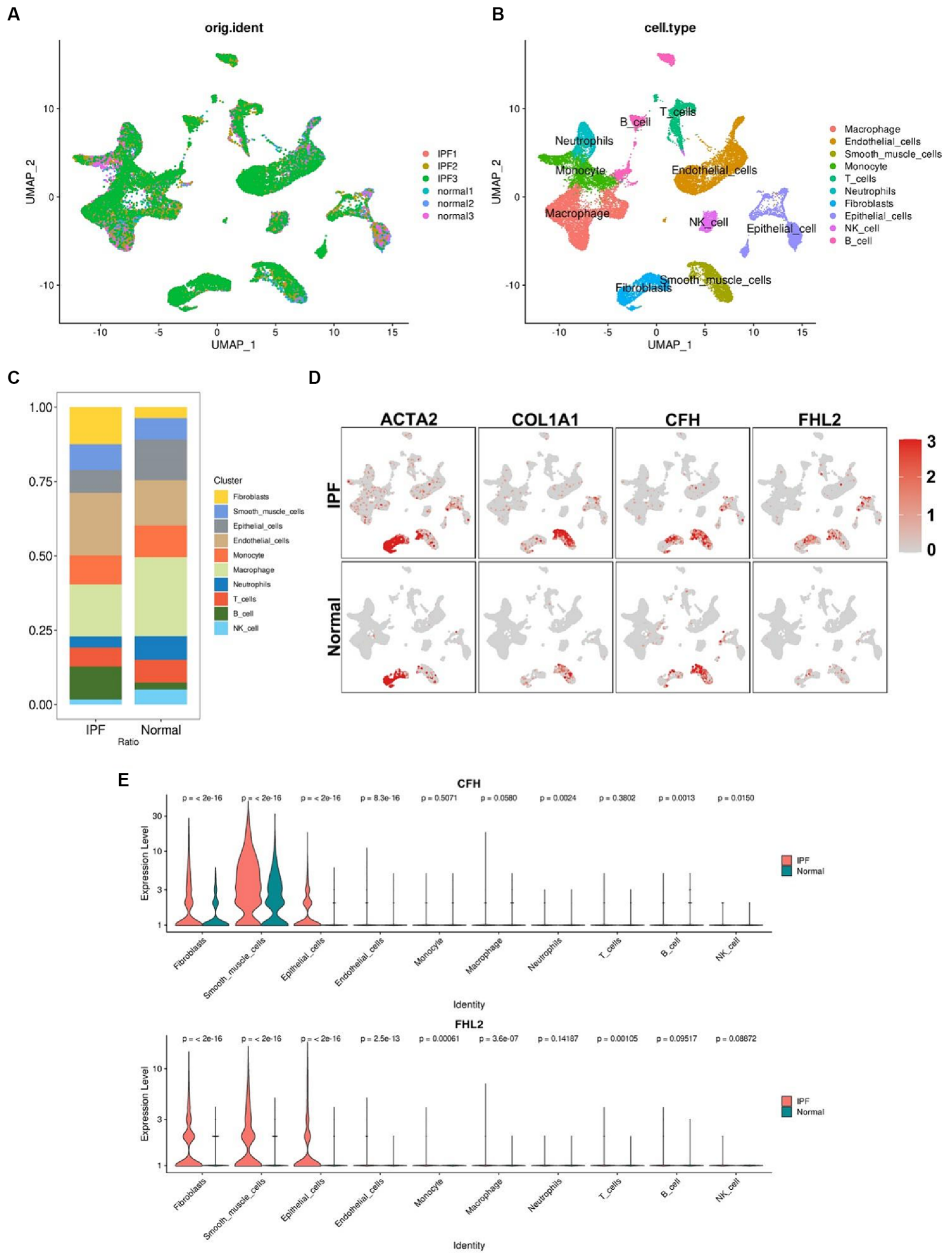
Single-cell RNA sequencing analysis. **(A)** Preprocessed single-cell data of GSE132771. **(B)** UMAP visualization of clustering revealing 10 cell clusters. **(C)** Comparison of the proportion of each cell cluster between IPF and normal group. The colors correspond to the cell types shown in the legend on the right. **(D)** Comparison of the expression levels of ACTA2, COL1A1, CFH, and FHL2 in different cell clusters between IPF and normal group. Red dots represent cells expressing the gene noted above. Shades of color correspond to the expression level shown in the legend on the right. **(E)** Violin plots compare the expression of CFH and FHL2 in different cell clusters between IPF and normal group with *p* value indicated above. CFH, complement factor H; FHL2, four and a half LIM domains 2; UMAP, Uniform Manifold Approximation and Projection.

We have more precisely localized the cell populations expressing CFH and FHL2 mRNA in IPF by single cell analysis. After a series of pre-processing of the data from the GSE132771, we performed a cluster analysis of the cells based on the similarity of the gene expression profile (Figures 8A,B). Among the 10 cell identities identified by clustering, the proportions of B cells, fibroblasts and endothelial cells in the IPF group were significantly increased, while epithelial cells, macrophages, neutrophils and NK cells were significantly decreased (Figure 8C). The expression levels of CFH and FHL2 in the IPF group were significantly upregulated in fibroblasts marked by ACTA2 and COL1A1, confirming that the two key genes obtained in this study are closely related to fibrosis induced by fibroblasts (*p* < 2^10–16) (Figures 8D,E).

### 3.8 Evaluation of CFH and FHL2 expression *in vivo* and *in vitro*

**Figure 9:**
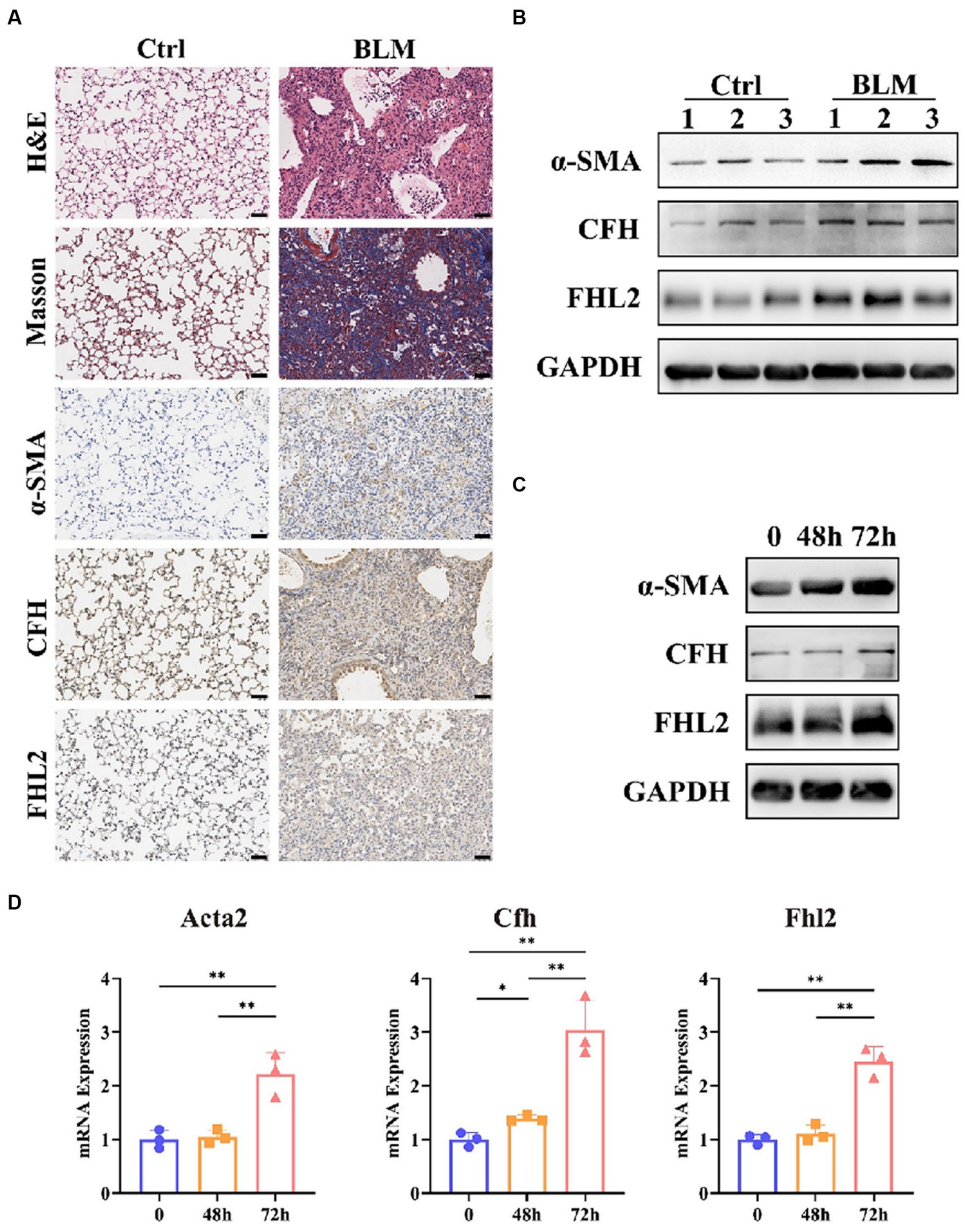
Expression of CFH and FHL2 in mouse and cellular IPF models. **(A)** HE and MASSON staining and immunohistochemical staining for α-SMA, CFH, and FHL2 in lung tissues of mice in control and bleomycin-induced groups. **(B)** Protein levels of α-SMA, CFH, and FHL2 in lung tissues of mice in control and bleomycin-induced groups assessed by Western blot. **(C)** Protein levels of α-SMA, CFH, and FHL2 in PLFs with or without TGF-β stimulation assessed by Western blot. **(D)** mRNA levels of Acta2, Cfh, and Fhl2 in PLFs with or without TGF-β stimulation assessed by qPCR. CFH, complement factor H; FHL2, four and a half LIM domains 2; α-SMA, alpha-smooth muscle actin; ACTA2, actin alpha 2, smooth muscle; PLFs, primary lung fibroblasts; TGFβ, transforming growth factor-β.

To verify the reliability of the results of bioinformatics analysis in IPF, we firstly established a IPF mouse model by bleomycin. H&E and Masson staining showed that 21 days after bleomycin administration, there was a significant increase in the deposition of collagen. Also, the expression of a widely accepted markers of fibroblast, α-smooth muscle actin (α-SMA) had a significant increase, as well as that of CFH and FHL2 (Figure 9A). WB analysis also showed that the content of α-SMA, CFH and FHL2 in the bleomycin-treated group increased compared with these in the control group (Figure 9B). Furthermore, we investigated whether CFH and FHL2 was involved in TGFβ1-induced fibroblasts activation. PLFs were treated with TGFβ1 (10 ng/mL) or PBS as control. And TGFβ1 treatment increased the expression of α-SMA, CFH and FHL2 both by WB analysis and qRT-PCR (Figures 9C,D). Collectively, these data indicated that CFH and FHL2 are involved in the pulmonary fibrosis and activation of fibroblasts induced by bleomycin and TGFβ1, respectively.

## 4 Discussion

Given that the underlying pathogenesis is not fully understood and the safe and effective therapeutic approaches remain elusive, currently there is an unmet clinical need for patients with IPF. In this study, we investigated the characteristics of the gene transcriptome in IPF patients compared to healthy individuals through comprehensive bioinformatics analysis, and identified a total of 422 up-regulated DEGs and 225 down-regulated DEGs. Functional enrichment analysis of overlapping DEGs revealed an enrichment of terms related to cell migration, structural organization, immune cell chemotaxis, extracellular matrix, etc., which is consistent with the existing literature. Notably, we further reduced the number of markers by taking the intersection of DEGs screened by the three machine learning algorithms, improving the specificity and sensitivity of the features of IPF. Through this process, CFH and FHL2 were eventually identified as key genes with significantly altered expression in the disease, and their accurate and safe diagnostic efficacy was confirmed by ROC curves and DCA, which was further supported by both *in vivo* and *in vitro* models.

Actin alpha 2, smooth muscle (ACTA2) encodes one of six different actin proteins (19). Collagen type I alpha 1 chain (COL1A1) encodes the pro-alpha1 chains of type I collagen whose triple helix comprises two alpha1 chains and one alpha2 chain (20). These two genes, ACTA2 and COL1A1, are currently considered to be the main markers of pathological fibrosis in IPF (21). However, their diagnostic specificity is limited due to the potential expression of these molecules in normal cells such as smooth muscle cells and pericytes, in addition to pathogenic fibroblasts that overproduce extracellular matrix (22). While in this study, the results of single-cell analysis revealed that the upregulation of CFH and FHL2 in the IPF group was predominantly localized in fibroblasts, suggesting that these two molecules may be able to serve as precise indicators of IPF fibroblast foci in IPF.

The human CFH gene is located on chromosome 1q32 within the RCA (complement activation regulation) gene cluster and encodes a 155 kDa glycoprotein called complement factor H (23). CFH protein consists of 20 short consensus repeats (SCRs) and is mainly synthesized by the liver before being secreted into the plasma. In addition to hepatic production, various cell types including myofibroblasts, peripheral blood lymphocytes, RPE cells, glomerular mesangial cells, and podocytes have been shown to express CFH extrahepatically, potentially contributing to localized concentration increases (24). CFH is an important negative regulator of complement alternative pathway (AP) through three distinct mechanisms: competitive binding to C3b with factor B to inhibit the formation of C3 convertase, displacement of C3b from the formed C3 convertase to accelerate the decay of complement activators, and functioning as a cofactor of factor I to facilitate the degradation and inactivation of C3b. Additionally, CFH binds autologous cells by interacting with sialic acid and heparin-like glycosaminoglycan polyanions on the surface of host cells, thereby shielding them from damage. The deficiency of CFH has been shown to be associated with a variety of diseases, such as membranoproliferative glomerulonephritis, atypical hemolytic uremic syndrome (aHUs), age-related macular degeneration (AMD), etc. (25–27). However, the relationship between CFH and IPF has not yet been reported. In this study, CFH was found to be highly expressed in lung fibroblasts from IPF samples by single-cell analysis and confirmed by experiments. Whether the change in the expression level of CFH is the result of inflammatory regulation disorder or a way for fibroblasts to protect themselves from immune damage needs to be further explored.

The FHL2 gene encodes a member of the four-and-a-half-LIM-only protein family, characterized by two highly conserved zinc finger domains, each with four cysteines bound to a zinc atom, which regulate transcription factor activity and cytoskeletal proteins. Due to its structural properties, FHL2 interacts with a wide range of proteins and participates in a variety of cellular processes, such as transcriptional regulation, cell differentiation, proliferation, migration, apoptosis, and signal transduction (28). Normally, FHL2 is only highly expressed in cardiac tissue, but as an early-response gene protein, FHL2 expression is upregulated during tissue remodeling. For example, FHL2 is difficult to detect in normal skin, but its expression is significantly increased during the repair process after skin injury, especially in the migration and proliferation phases. Notably, FHL2 is predominantly expressed in myofibroblasts during this period, indicating a close association with its functional role. It stimulates fibroblast migration in a RAC-dependent manner, regulates matrix assembly, and acts as a transcriptional cofactor to support the expression of α-SMA and ECM proteins (29–33). Consequently, elevated levels of FHL2 may be implicated in the excessive wound healing and tissue remodeling observed in patients with IPF. Several studies have indicated that increased FHL2 expression could potentially serve as a distinguishing factor between individuals with IPF and healthy controls (34). The expression level of FHL2 was significantly and negatively correlated with percent diffusing capacity of the lungs for carbon monoxide (%DLCO), suggesting a potential role for FHL2 in stratifying patients based on disease severity (35). Experiments have shown that FHL2 inhibitors can significantly delay the progression of pulmonary fibrosis (36). Nevertheless, the relationship between FHL2 and immune dysregulation in IPF disease states has not been reported.

The relationship between IPF and immunity or inflammation is currently unclear. Some views suggest that aberrant immune activation plays a role in the development of IPF, while others argue that inflammation is a secondary characteristic of the disease, as evidenced by the limited efficacy of anti-inflammatory treatments in clinical trials. In this study, many DEGs in IPF samples were found to be associated with the chemotaxis and migration of a variety of immune cells as well as with the activity of immune receptors by functional enrichment analysis, confirming the close relationship between IPF and immunity. Further analysis showed that FHL2 and CFH were significantly positively correlated with B cells, and B cells were significantly enriched in IPF lung samples, suggesting a potential role for these genes in regulating B cell function in IPF. Previous studies have suggested that the binding of CFH and/or its related proteins to B lymphocytes may influence the migration of these cells and their role in adaptive immunity, further supporting the findings of this study (24, 37–39). Regarding FHL2, it is involved in the regulation of immune cell infiltration through direct interaction with various integrins, particularly the B2 subunit of the CD11a-d and CD18 integrin heterodimer receptor on immune cells. Additionally, FHL2 may indirectly influence immune cell attraction by modulating the expression of pro-inflammatory or anti-inflammatory cytokines (40).

This study also has some limitations. Firstly, the utilization of datasets from a public database without comprehensive clinical information hindered the ability to conduct prognostic analyses to ascertain the correlation between aberrant alterations in biomarker expression and poor patient prognosis. Further clinical studies are necessary to validate the diagnostic and stratification efficacy of biomarkers. Furthermore, the molecular mechanism of biomarkers in the pathogenesis of IPF and their relationship with immunity have not been elucidated. Additional research is required to investigate and identify potential therapeutic targets for IPF.

In summary, CFH and FHL2 have been identified as promising novel biomarkers for IPF with strong diagnostic capabilities, suggesting their potential utility as diagnostic aids in the future. Further studies of the role of these two genes in IPF and their relationship with immune cells could help to understand the pathogenesis of IPF and provide potential therapeutic targets.

## Data availability statement

The datasets presented in this study can be found in online repositories. The names of the repository/repositories and accession number(s) can be found below: https://www.ncbi.nlm.nih.gov/geo/, GSE150910 https://www.ncbi.nlm.nih.gov/geo/, GSE32537 https://www.ncbi.nlm.nih.gov/geo/, GSE132771.

## Ethics statement

The animal study was approved by Committee on Ethics of Medicine, Navy Medical University, PLA. The study was conducted in accordance with the local legislation and institutional requirements.

## Author contributions

XL: Conceptualization, Data curation, Formal analysis, Investigation, Methodology, Project administration, Software, Validation, Writing – original draft, Writing – review & editing. MY: Conceptualization, Data curation, Investigation, Methodology, Validation, Writing – original draft, Writing – review & editing. JL: Data curation, Formal analysis, Investigation, Validation, Writing – original draft, Writing – review & editing. HL: Methodology, Software, Supervision, Writing – original draft, Writing – review & editing. YD: Conceptualization, Funding acquisition, Resources, Supervision, Validation, Writing – original draft, Writing – review & editing. JZ: Conceptualization, Funding acquisition, Supervision, Validation, Visualization, Writing – original draft, Writing – review & editing. YH: Conceptualization, Funding acquisition, Investigation, Methodology, Resources, Supervision, Validation, Writing – original draft, Writing – review & editing.
