# Supplementary material for: Identification of CFH and FHL2 as biomarkers for idiopathic pulmonary fibrosis
Source: Front Med (Lausanne). 2024 May 9;11:1363643. doi: 10.3389/fmed.2024.1363643 (PMC11111937; doi:10.3389/fmed.2024.1363643)
Supplement: Supplementary file 2 [file Table_2.DOCX]

#count转化为TPM

#读入length

setwd("~") #设置工作目录

len=read.table('All_hg19gene_len.txt',header = T,sep = '\t')

colnames(len)[1] = 'SYMBOL'

# read expression Matter

exp=read.csv('GSE150910_gene-level_count_file.csv')

head(exp)

#链接

library(dplyr)

merge<-left_join(exp,len,by="SYMBOL")#根据基因那列进行合并

merge <- na.omit(merge)#删除错误值行

write.csv(merge,file = "merge.csv",sep = "\t")#读出文件，直接往下运行也许

#计算TPM

head(merge)

merge1 <- merge[!duplicated(merge$SYMBOL),]

rownames(merge1)<-merge1$SYMBOL

merge1<-merge1[,-1]

head(merge1)#最后一列Length是基因长度

kb <- merge1$Length/1000

kb

countdata <- merge1[,1:289]#289是总列数

rpk <- countdata / kb

rpk

tpm <- t(t(rpk)/colSums(rpk) * 1000000)

head(tpm)

write.table(tpm,file="tpm.xls",sep="\t",quote=F)

#判断数据是否需要log2转化

#引用包

library(limma)

library(pheatmap)

library(sva)

setwd("~/liuxingchen/pancancer") #设置工作目录

inputFile="symbol.LIHC.txt" #输入文件

#读取输入文件，并对输入文件整理

rt=read.table(inputFile, header=T, sep="\t", check.names=F)

rt=as.matrix(rt)

rownames(rt)=rt[,1]

exp=rt[,2:ncol(rt)]

dimnames=list(rownames(exp),colnames(exp))

data=matrix(as.numeric(as.matrix(exp)),nrow=nrow(exp),dimnames=dimnames)

rt=avereps(data)

#对数值大的数据取log2(TPM+1)

qx=as.numeric(quantile(rt, c(0, 0.25, 0.5, 0.75, 0.99, 1.0), na.rm=T))

LogC=(qx[5]>100) || (qx[6]-qx[1]>50 && qx[2]>0) ||(qx[2]>0&&qx[2]<1&&qx[4]>1&&qx[4]<2)

if(LogC){

rt[which(rt<=0)]<-NaN

rt1=log2(rt+1)

print("log2 transform finished")}else{print("log2 transform not")}

rt=avereps(data)

rt1=log2(rt+1)

write.table(rt1, file="rt1.txt", sep="\t", quote=F, col.names=T)

#02diff进行limma差异分析

library(limma)

library(pheatmap)

inputFile="GSE32537.txt" #输入文件

logFCfilter=0.585 #logFC过滤阈值

adj.P.Val.Filter=0.05 #矫正后p值阈值

setwd("~/liuxingchen/YMIPF/GEO/02diff/GSE32537") #设置工作目录

#读取输入文件，并对输入文件整理

rt=read.table(inputFile, header=T, sep="\t", check.names=F)

rt=as.matrix(rt)

rownames(rt)=rt[,1]

exp=rt[,2:ncol(rt)]

dimnames=list(rownames(exp),colnames(exp))

data=matrix(as.numeric(as.matrix(exp)),nrow=nrow(exp),dimnames=dimnames)

data=avereps(data)

data=data[rowMeans(data)>0,]

#读取目录下所有"s1.txt"结尾的文件

sampleName1=c()

files=dir()

files=grep("s1.txt$", files, value=T)

for(file in files){

rt=read.table(file, header=F, sep="\t", check.names=F) #读取输入文件

geneNames=as.vector(rt[,1]) #提取基因名称

uniqGene=unique(geneNames) #基因取unique

sampleName1=c(sampleName1, uniqGene)

}

#读取目录下所有"s2.txt"结尾的文件

sampleName2=c()

files=dir()

files=grep("s2.txt$", files, value=T)

for(file in files){

rt=read.table(file, header=F, sep="\t", check.names=F) #读取输入文件

geneNames=as.vector(rt[,1]) #提取基因名称

uniqGene=unique(geneNames) #基因取unique

sampleName2=c(sampleName2, uniqGene)

}

#提取实验组和对照组的数据

conData=data[,sampleName1]

treatData=data[,sampleName2]

data=cbind(conData,treatData)

conNum=ncol(conData)

treatNum=ncol(treatData)

#差异分析

Type=c(rep("con",conNum),rep("treat",treatNum))

design <- model.matrix(~0+factor(Type))

colnames(design) <- c("con","treat")

fit <- lmFit(data,design)

cont.matrix<-makeContrasts(treat-con,levels=design)

fit2 <- contrasts.fit(fit, cont.matrix)

fit2 <- eBayes(fit2)

allDiff=topTable(fit2,adjust='fdr',number=200000)

allDiffOut=rbind(id=colnames(allDiff),allDiff)

write.table(allDiffOut, file="all.txt", sep="\t", quote=F, col.names=F)

#输出矫正后的表达量

outData=rbind(id=paste0(colnames(data),"_",Type),data)

write.table(outData, file="normalize.txt", sep="\t", quote=F, col.names=F)

#输出差异结果

diffSig=allDiff[with(allDiff, (abs(logFC)>logFCfilter & adj.P.Val < adj.P.Val.Filter )), ]

diffSigOut=rbind(id=colnames(diffSig),diffSig)

write.table(diffSigOut, file="diff.txt", sep="\t", quote=F, col.names=F)

#输出差异基因表达量

diffGeneExp=data[row.names(diffSig),]

diffGeneExpOut=rbind(id=paste0(colnames(diffGeneExp),"_",Type), diffGeneExp)

write.table(diffGeneExpOut, file="diffGeneExp.txt", sep="\t", quote=F, col.names=F)

#绘制差异基因热图

geneNum=25

diffSig=diffSig[order(as.numeric(as.vector(diffSig$logFC))),]

diffGeneName=as.vector(rownames(diffSig))

diffLength=length(diffGeneName)

hmGene=c()

if(diffLength>(2*geneNum)){

hmGene=diffGeneName[c(1:geneNum,(diffLength-geneNum+1):diffLength)]

}else{

hmGene=diffGeneName

}

hmExp=data[hmGene,]

Type=c(rep("Normal",conNum),rep("IPF",treatNum))

names(Type)=colnames(data)

Type=as.data.frame(Type)

pdf(file="heatmap.pdf", width=8, height=8.5)

pheatmap(hmExp,

annotation=Type,

color = colorRampPalette(c("blue", "white", "red"))(50),

cluster_cols =F,

show_colnames = F,

scale="row",

fontsize = 12,

fontsize_row=12,

fontsize_col=12)

dev.off()

#03火山图

library(ggplot2)

library(tidyverse)

library(ggrepel) #引用包

logFCfilter=0.585 #logFC过滤条件

adj.P.Val.Filter=0.05 #矫正后的p值过滤条件

inputFile="all.txt" #输入文件

setwd("~/liuxingchen/YMIPF/GEO/03volcano/GSE32537") #设置工作目录

#读取输入文件

rt=read.table(inputFile, header=T, sep="\t", check.names=F)

#定义显著性

Sig=ifelse((rt$adj.P.Val<adj.P.Val.Filter) & (abs(rt$logFC)>logFCfilter), ifelse(rt$logFC>logFCfilter,"Up","Down"), "Not")

rt=cbind(rt, Sig=Sig)

#绘制火山图不带标签

p=ggplot(rt, aes(logFC, -log10(adj.P.Val)))+

geom_point(aes(col=Sig))+

scale_color_manual(values=c("blue", "gray", "red"))+

xlim(-4,4)+

labs(title = " ")+

geom_vline(xintercept=c(-logFCfilter,logFCfilter), col="dimgray", cex=1, linetype=2)+

geom_hline(yintercept= -log10(adj.P.Val.Filter), col="dimgray", cex=1, linetype=2)+

theme(plot.title=element_text(size=16, hjust=0.5, face="bold"))

p=p+theme_bw()

#绘制火山图带有标签特定基因

p=ggplot(rt, aes(logFC, -log10(adj.P.Val)))+

geom_point(aes(col=Sig))+

scale_color_manual(values=c("green", "black", "red"))+

xlim(-5,5)+

labs(title = " ")+

geom_vline(xintercept=c(-logFCfilter,logFCfilter), col="blue", cex=1, linetype=2)+

geom_hline(yintercept= -log10(adj.P.Val.Filter), col="blue", cex=1, linetype=2)+

geom_text_repel(data=rt %>%

filter(id %in% c("ECM1","LIFR","SRPX","TBXA2R")),

aes(label=id),size=3,color="black",box.padding=unit(0.5, "lines"),

point.padding=NA, segment.color = "black", show.legend = FALSE) +

theme(plot.title=element_text(size=16, hjust=0.5, face="bold"))

p=p+theme_bw()

#输出火山图

pdf(file="volcano.pdf", width=5, height=6)

print(p)

dev.off()

#机器学习算法WGCNA

#引用包WGCNA

rm(list=ls())

library(limma)

library(WGCNA)

library(stringr)

expFile="normalize.txt" #表达数据文件

setwd("~/liuxingchen/YMIPF/GEO/06WGCNA/GSE150910") #设置工作目录

#读取输入文件，并对输入文件整理

rt=read.table(expFile, header=T, sep="\t", check.names=F)

rt=as.matrix(rt)

rownames(rt)=rt[,1]

exp=rt[,2:ncol(rt)]

dimnames=list(rownames(exp),colnames(exp))

data=matrix(as.numeric(as.matrix(exp)),nrow=nrow(exp),dimnames=dimnames)

data=avereps(data)

#data=data[apply(data,1,sd)>0.7,] #删除波动小的基因

#查看具体结构

dim(data)

length(colnames(data))

#提取对照组和实验组的样品信息

Type=gsub("(.*)\\_(.*)", "\\2", colnames(data))

Normal=length(Type[Type=="con"])

Tumor=length(Type[Type=="treat"])

datExpr0=t(data)

###检查缺失值

gsg = goodSamplesGenes(datExpr0, verbose = 3)

if (!gsg$allOK)

{

# Optionally, print the gene and sample names that were removed:

if (sum(!gsg$goodGenes)>0)

printFlush(paste("Removing genes:", paste(names(datExpr0)[!gsg$goodGenes], collapse = ", ")))

if (sum(!gsg$goodSamples)>0)

printFlush(paste("Removing samples:", paste(rownames(datExpr0)[!gsg$goodSamples], collapse = ", ")))

# Remove the offending genes and samples from the data:

datExpr0 = datExpr0[gsg$goodSamples, gsg$goodGenes]

}

###样品聚类

sampleTree = hclust(dist(datExpr0), method = "average")

pdf(file = "1_sample_cluster.pdf", width = 12, height = 9)

par(cex = 0.6)

par(mar = c(0,4,2,0))

plot(sampleTree, main = "Sample clustering to detect outliers", sub="", xlab="", cex.lab = 1.5, cex.axis = 1.5, cex.main = 2)

###剪切线

abline(h = 135, col = "red")

dev.off()

###删除剪切线以下的样品

clust = cutreeStatic(sampleTree, cutHeight = 135, minSize = 10)

table(clust)

keepSamples = (clust==1)

datExpr0 = datExpr0[keepSamples, ]

###准备临床数据

traitData=data.frame(Con=c(rep(1,Normal),rep(0,Tumor)),

Treat=c(rep(0,Normal),rep(1,Tumor)))

row.names(traitData)=colnames(data)

tpmSamples = rownames(datExpr0)

traitSamples =rownames(traitData)

sameSample=intersect(tpmSamples,traitSamples)

datExpr0=datExpr0[sameSample,]

datTraits=traitData[sameSample,]

###样品聚类,得到样品聚类的热图

sampleTree2 = hclust(dist(datExpr0), method = "average")

traitColors = numbers2colors(datTraits, signed = FALSE)

pdf(file="2_sample_heatmap.pdf",width=12,height=12)

plotDendroAndColors(sampleTree2, traitColors,

groupLabels = names(datTraits),

main = "Sample dendrogram and trait heatmap")

dev.off()

###power值散点图

enableWGCNAThreads() #多线程工作

powers = c(1:20) #幂指数范围1:20

sft = pickSoftThreshold(datExpr0, powerVector = powers, verbose = 5)

pdf(file="3_scale_independence.pdf",width=9,height=5)

par(mfrow = c(1,2))

cex1 = 0.9

###拟合指数与power值散点图

plot(sft$fitIndices[,1], -sign(sft$fitIndices[,3])*sft$fitIndices[,2],

xlab="Soft Threshold (power)",ylab="Scale Free Topology Model Fit,signed R^2",type="n",

main = paste("Scale independence"));

text(sft$fitIndices[,1], -sign(sft$fitIndices[,3])*sft$fitIndices[,2],

labels=powers,cex=cex1,col="red");

abline(h=0.90,col="red") #可以修改

###平均连通性与power值散点图

plot(sft$fitIndices[,1], sft$fitIndices[,5],

xlab="Soft Threshold (power)",ylab="Mean Connectivity", type="n",

main = paste("Mean connectivity"))

text(sft$fitIndices[,1], sft$fitIndices[,5], labels=powers, cex=cex1,col="red")

dev.off()

###邻接矩阵转换

sft #查看最佳power值

softPower =sft$powerEstimate #最佳power值

adjacency = adjacency(datExpr0, power = softPower)

softPower

#无尺度网络的拓扑关系

pdf(file="3_softConnectivity.pdf", width=9, height=5)

k <- softConnectivity(datE=datExpr0,power=softPower)

#sizeGrWindow(10, 5)

par(mfrow=c(1,2))

hist(k)

scaleFreePlot(k,main="Check Scale free topology\n")

dev.off()

###TOM矩阵

TOM = TOMsimilarity(adjacency)

dissTOM = 1-TOM

###基因聚类

geneTree = hclust(as.dist(dissTOM), method = "average");

pdf(file="4_gene_clustering.pdf",width=12,height=9)

plot(geneTree, xlab="", sub="", main = "Gene clustering on TOM-based dissimilarity",

labels = FALSE, hang = 0.04)

dev.off()

###动态剪切模块识别

minModuleSize = 60 #设定最小模块基因数目40/50/60

dynamicMods = cutreeDynamic(dendro = geneTree, distM = dissTOM,

deepSplit = 2, pamRespectsDendro = FALSE,

minClusterSize = minModuleSize);

table(dynamicMods) #查看得到的模块数量

dynamicColors = labels2colors(dynamicMods)

table(dynamicColors)

pdf(file="5_Dynamic_Tree.pdf",width=8,height=6)

plotDendroAndColors(geneTree, dynamicColors, "Dynamic Tree Cut",

dendroLabels = FALSE, hang = 0.03,

addGuide = TRUE, guideHang = 0.05,

main = "Gene dendrogram and module colors")

dev.off()

###对模块进行聚类,找出相似模块聚类

MEList = moduleEigengenes(datExpr0, colors = dynamicColors)

MEs = MEList$eigengenes

MEDiss = 1-cor(MEs);

METree = hclust(as.dist(MEDiss), method = "average")

pdf(file="6_Clustering_module.pdf",width=7,height=6)

plot(METree, main = "Clustering of module eigengenes",

xlab = "", sub = "")

MEDissThres = 0.4 #剪切高度可修改

abline(h=MEDissThres, col = "red")

dev.off()

###相似模块合并

merge = mergeCloseModules(datExpr0, dynamicColors, cutHeight = MEDissThres, verbose = 3)

mergedColors = merge$colors

mergedMEs = merge$newMEs

pdf(file="7_merged_dynamic.pdf", width = 8, height = 5)

plotDendroAndColors(geneTree, mergedColors,"Dynamic Tree Cut",

dendroLabels = FALSE, hang = 0.03,

addGuide = TRUE, guideHang = 0.05,

main = "Gene dendrogram and module colors")

dev.off()

moduleColors = mergedColors

table(moduleColors)

colorOrder = c("grey", standardColors(50))

moduleLabels = match(moduleColors, colorOrder)-1

MEs = mergedMEs

###模块与性状数据热图

nGenes = ncol(datExpr0)

nSamples = nrow(datExpr0)

moduleTraitCor = cor(MEs, datTraits, use = "p")

moduleTraitPvalue = corPvalueStudent(moduleTraitCor, nSamples)

pdf(file="8_Module_trait.pdf", width=7, height=5)

textMatrix = paste(signif(moduleTraitCor, 2), "\n(",

signif(moduleTraitPvalue, 1), ")", sep = "")

dim(textMatrix) = dim(moduleTraitCor)

par(mar = c(5, 10, 3, 3))

labeledHeatmap(Matrix = moduleTraitCor,

xLabels = names(datTraits),

yLabels = names(MEs),

ySymbols = names(MEs),

colorLabels = FALSE,

colors = blueWhiteRed(50),

textMatrix = textMatrix,

setStdMargins = FALSE,

cex.text = 0.8,

zlim = c(-1,1),

main = paste("Module-trait relationships"))

dev.off()

###计算MM和GS值

modNames = substring(names(MEs), 3)

geneModuleMembership = as.data.frame(cor(datExpr0, MEs, use = "p"))

MMPvalue = as.data.frame(corPvalueStudent(as.matrix(geneModuleMembership), nSamples))

names(geneModuleMembership) = paste("MM", modNames, sep="")

names(MMPvalue) = paste("p.MM", modNames, sep="")

traitNames=names(datTraits)

geneTraitSignificance = as.data.frame(cor(datExpr0, datTraits, use = "p"))

GSPvalue = as.data.frame(corPvalueStudent(as.matrix(geneTraitSignificance), nSamples))

names(geneTraitSignificance) = paste("GS.", traitNames, sep="")

names(GSPvalue) = paste("p.GS.", traitNames, sep="")

###输出模块重要性的图形

y=datTraits[,1]

GS1=as.numeric(cor(y, datExpr0, use="p"))

GeneSignificance=abs(GS1)

ModuleSignificance=tapply(GeneSignificance, mergedColors, mean, na.rm=T)

pdf(file="9_GeneSignificance.pdf", width=11, height=7)

plotModuleSignificance(GeneSignificance, mergedColors)

dev.off()

###批量输出性状和模块散点图

trait="Treat"

traitColumn=match(trait,traitNames)

for (module in modNames){

column = match(module, modNames)

moduleGenes = moduleColors==module

if (nrow(geneModuleMembership[moduleGenes,]) > 1){

outPdf=paste("10_", trait, "_", module,".pdf",sep="")

pdf(file=outPdf,width=7,height=7)

par(mfrow = c(1,1))

verboseScatterplot(abs(geneModuleMembership[moduleGenes, column]),

abs(geneTraitSignificance[moduleGenes, traitColumn]),

xlab = paste("Module Membership in", module, "module"),

ylab = paste("Gene significance for Type"),

main = paste("Module membership vs. gene significance\n"),

cex.main = 1.2, cex.lab = 1.2, cex.axis = 1.2, col = module)

abline(v=0.8,h=0.5,col="red")

dev.off()

}

}

###输出GS_MM数据

probes = colnames(datExpr0)

geneInfo0 = data.frame(probes= probes,

moduleColor = moduleColors)

for (Tra in 1:ncol(geneTraitSignificance))

{

oldNames = names(geneInfo0)

geneInfo0 = data.frame(geneInfo0, geneTraitSignificance[,Tra],

GSPvalue[, Tra])

names(geneInfo0) = c(oldNames,names(geneTraitSignificance)[Tra],

names(GSPvalue)[Tra])

}

for (mod in 1:ncol(geneModuleMembership))

{

oldNames = names(geneInfo0)

geneInfo0 = data.frame(geneInfo0, geneModuleMembership[,mod],

MMPvalue[, mod])

names(geneInfo0) = c(oldNames,names(geneModuleMembership)[mod],

names(MMPvalue)[mod])

}

geneOrder =order(geneInfo0$moduleColor)

geneInfo = geneInfo0[geneOrder, ]

write.table(geneInfo, file = "GS_MM.xls",sep="\t",row.names=F)

###输出每个模块的基因

for (mod in 1:nrow(table(moduleColors)))

{

modules = names(table(moduleColors))[mod]

probes = colnames(datExpr0)

inModule = (moduleColors == modules)

modGenes = probes[inModule]

write.table(modGenes, file =paste0("module_",modules,".txt"),sep="\t",row.names=F,col.names=F,quote=F)

}

###输出每个模块的核心基因

geneSigFilter=0.5 #基因重要性的过滤条件

moduleSigFilter=0.8 #基因与模块相关性的过滤条件

datMM=cbind(geneModuleMembership, geneTraitSignificance)

datMM=datMM[abs(datMM[,ncol(datMM)])>geneSigFilter,]

for(mmi in colnames(datMM)[1:(ncol(datMM)-2)]){

dataMM2=datMM[abs(datMM[,mmi])>moduleSigFilter,]

write.table(row.names(dataMM2), file =paste0("hubGenes_",mmi,".txt"),sep="\t",row.names=F,col.names=F,quote=F)

}

#机器学习算法Lasso

#引用包

rm(list=ls())

set.seed(111)

library(glmnet) #引用包

expFile="normalize.txt" #表达数据文件

geneFile="interGenes.txt" #基因列表文件

setwd("~/liuxingchen/YMIPF/GEO/05LASSO/GSE32537") #设置工作目录

#读取表达数据文件

rt=read.table(expFile, header=T, sep="\t", check.names=F, row.names=1)

#读取交集基因列表文件,提取交集基因的表达量

geneRT=read.table(geneFile, header=F, sep="\t", check.names=F)

rt=rt[as.vector(geneRT[,1]),]

rt=t(rt)

#构建Lasso回归模型

x=as.matrix(rt)

y=gsub("(.*)\\_(.*)", "\\2", row.names(rt))

fit=glmnet(x, y, family = "binomial", alpha=1)

#绘制Lasso回归的图形

pdf(file="lasso.pdf", width=6, height=5.5)

plot(fit)

dev.off()

#绘制交叉验证的图形

cvfit=cv.glmnet(x, y, family="binomial", alpha=1,type.measure='deviance',nfolds = 10)

pdf(file="cvfit.pdf", width=6, height=5.5)

plot(cvfit)

dev.off()

#输出疾病的特征基因

coef=coef(fit, s = cvfit$lambda.min)

index=which(coef != 0)

lassoGene=row.names(coef)[index]

lassoGene=lassoGene[-1]

write.table(lassoGene, file="LASSO.gene.txt", sep="\t", quote=F, row.names=F, col.names=F)

#机器学习算法--RF随机森林法

rm(list=ls())

library(randomForest)

set.seed(111)

inputFile="diffGeneExp.txt" #输入文件

setwd("~/liuxingchen/YMIPF/GEO/04RF/GSE32537") #设置工作目录

#读取输入文件

data=read.table(inputFile, header=T, sep="\t", check.names=F, row.names=1)

data=t(data)

group=gsub("(.*)\\_(.*)", "\\2", row.names(data))

#随机森林树

rf=randomForest(as.factor(group)~., data=data, ntree=500)

pdf(file="forest.pdf", width=6, height=6)

plot(rf, main="Random forest", lwd=2)

dev.off()

#找出误差最小的点

optionTrees=which.min(rf$err.rate[,1])

optionTrees

rf2=randomForest(as.factor(group)~., data=data, ntree=optionTrees)

#查看基因的重要性

importance=importance(x=rf2)

#绘制基因的重要性图

pdf(file="geneImportance.pdf", width=3.5, height=6)

varImpPlot(rf2, main="")

dev.off()

#挑选疾病特征基因

rfGenes=importance[order(importance[,"MeanDecreaseGini"], decreasing = TRUE),]

rfGenes=names(rfGenes[rfGenes>1]) #挑选重要性评分大于1的基因

#rfGenes=names(rfGenes[1:5]) #挑选重要性评分最高的5个基因

write.table(rfGenes, file="rfGenes.txt", sep="\t", quote=F, col.names=F, row.names=F)

#输出重要基因的表达量

sigExp=t(data[,rfGenes])

sigExpOut=rbind(ID=colnames(sigExp),sigExp)

write.table(sigExpOut, file="rfGeneExp.txt", sep="\t", quote=F, col.names=F)

#机器学习算法--SVM-RFE

rm(list=ls())

library(e1071)

library(kernlab)

library(caret)

set.seed(111)

inputFile="diffGeneExp.txt" #输入文件

setwd("~/liuxingchen/YMIPF/GEO/07SVMRFE/GSE32537") #设置工作目录

#读取输入文件

data=read.table(inputFile, header=T, sep="\t", check.names=F, row.names=1)

data=t(data)

group=gsub("(.*)\\_(.*)", "\\2", row.names(data))

#SVM-RFE分析

Profile=rfe(x=data,

y=as.numeric(as.factor(group)),

sizes = c(2,4,6,8, seq(10,40,by=3)),

rfeControl = rfeControl(functions = caretFuncs, method = "cv"),

methods="svmRadial")

#绘制图形

pdf(file="SVM-RFE.pdf", width=6, height=5.5)

par(las=1)

x = Profile$results$Variables

y = Profile$results$RMSE

plot(x, y, xlab="Variables", ylab="RMSE (Cross-Validation)", col="darkgreen")

lines(x, y, col="darkgreen")

#标注交叉验证误差最小的点

wmin=which.min(y)

wmin.x=x[wmin]

wmin.y=y[wmin]

points(wmin.x, wmin.y, col="blue", pch=16)

text(wmin.x, wmin.y, paste0('N=',wmin.x), pos=2, col=2)

dev.off()

#输出选择的基因

featureGenes=Profile$optVariables

write.table(file="SVM-RFE.gene.txt", featureGenes, sep="\t", quote=F, row.names=F, col.names=F)

#核心基因表达分析

rm(list=ls())

library(limma)

library(reshape2)

library(ggpubr)

expFile="GSE32537.txt" #表达数据文件

conFile="GSE32537_s1.txt" #对照组样品信息文件

treatFile="GSE32537_s2.txt" #实验组样品信息文件

geneFile="interGenes.txt" #基因列表文件

setwd("~/liuxingchen/YMIPF/GEO/09Expression/GSE32537") #设置工作目录

#读取输入文件

rt=read.table(expFile, header=T, sep="\t", check.names=F)

rt=as.matrix(rt)

rownames(rt)=rt[,1]

exp=rt[,2:ncol(rt)]

dimnames=list(rownames(exp), colnames(exp))

data=matrix(as.numeric(as.matrix(exp)), nrow=nrow(exp), dimnames=dimnames)

rt=avereps(data)

#对数值大的数据取log2

qx=as.numeric(quantile(rt, c(0, 0.25, 0.5, 0.75, 0.99, 1.0), na.rm=T))

LogC=( (qx[5]>100) || ( (qx[6]-qx[1])>50 && qx[2]>0) )

if(LogC){

rt[rt<0]=0

rt=log2(rt+1)}

data=normalizeBetweenArrays(rt)

#判断样品类型

Normal=read.table(conFile, header=F, sep="\t", check.names=F)

IPF=read.table(treatFile, header=F, sep="\t", check.names=F)

NormalData=data[,as.vector(Normal[,1])]

IPFData=data[,as.vector(IPF[,1])]

data=cbind(NormalData, IPFData)

NormalNum=ncol(NormalData)

IPFNum=ncol(IPFData)

#输出矫正后的表达量

Type=c(rep("Normal",NormalNum), rep("IPF",IPFNum))

outData=rbind(id=paste0(colnames(data),"_",Type),data)

write.table(outData, file="test.normalize.txt", sep="\t", quote=F, col.names=F)

#读取交集基因的文件,提取目标基因表达量

geneRT=read.table(geneFile, header=F, sep="\t", check.names=F)

data=data[as.vector(geneRT[,1]),,drop=F]

#设置比较组

Type=c(rep("Normal",NormalNum), rep("IPF",IPFNum))

my_comparisons=list()

my_comparisons[[1]]=levels(factor(Type))

#差异分析

newGeneLists=c()

outTab=data.frame()

for(i in row.names(data)){

#data[i,][data[i,]>quantile(data[i,], 0.99)]=quantile(data[i,], 0.99)

rt1=data.frame(expression=data[i,], Type=Type)

#对差异基因进行可视化，绘制箱线图

boxplot=ggboxplot(rt1, x="Type", y="expression", color="Type",

xlab="",

ylab=paste(i, "expression"),

legend.title="",

palette = c("blue", "red"),

add = "jitter")+

stat_compare_means(comparisons = my_comparisons)

#输出图片

pdf(file=paste0("boxplot.",i,".pdf"), width=5, height=4.5)

print(boxplot)

dev.off()

}

#08PCA分析

#引用包

library(limma)

library(ggplot2)

expFile="GSE32537.txt" #表达数据文件

clusterFile="cluster.txt" #分型的结果文件

setwd("~/liuxingchen/YMIPF/GEO/08PCA/32537") #设置工作目录

#读取输入文件,并对输入文件进行整理

rt=read.table(expFile, header=T, sep="\t", check.names=F)

rt=as.matrix(rt)

rownames(rt)=rt[,1]

exp=rt[,2:ncol(rt)]

dimnames=list(rownames(exp),colnames(exp))

data=matrix(as.numeric(as.matrix(exp)),nrow=nrow(exp),dimnames=dimnames)

data=avereps(data)

data=data[rowMeans(data)>0,]

data=t(data)

#PCA分析

#PCA分析发现报错cannot rescale a constant/zero column to unit variance

#PCA分析which(apply(data, 2, var)==0)

#PCA分析data=data[ , which(apply(data, 2, var) != 0)]

data.pca=prcomp(data, scale. = TRUE)

pcaPredict=predict(data.pca)

write.table(pcaPredict, file="PCA.result.txt", quote=F, sep="\t")

#读取分型文件

cluster=read.table(clusterFile, header=T, sep="\t", check.names=F, row.names=1)

CRGcluster=as.vector(cluster[,1])

#设置分型的颜色

bioCol=c("#0066FF","#FF9900","#FF0000","#6E568C","#7CC767","#223D6C","#D20A13","#FFD121","#088247","#11AA4D")

crgCluCol=bioCol[1:length(levels(factor(CRGcluster)))]

#绘制图形

PCA=data.frame(PC1=pcaPredict[,1], PC2=pcaPredict[,2], CRGcluster=CRGcluster)

PCA.mean=aggregate(PCA[,1:2], list(CRGcluster=PCA$CRGcluster), mean)

pdf(file="PCA.pdf", width=6, height=4.6)

ggplot(data = PCA, aes(PC1, PC2)) + geom_point(aes(color = CRGcluster)) +

scale_colour_manual(name="Type", values =crgCluCol)+

theme_bw()+

theme(plot.margin=unit(rep(1.5,4),'lines'))+

annotate("text",x=PCA.mean$PC1, y=PCA.mean$PC2, label=PCA.mean$CRGcluster, cex=7)+

theme(panel.grid.major = element_blank(), panel.grid.minor = element_blank())

dev.off()

#10ssGSEA

library(reshape2)

library(ggpubr)

library(limma)

library(GSEABase)

library(GSVA)

expFile="normalize.txt" #表达数据文件

gmtFile="immune.gmt" #免疫基因集文件

setwd("~/liuxingchen/YMIPF/GEO/10ssGSEA/150910") #设置工作目录

#读取表达输入文件,并对输入文件整理

rt=read.table(expFile, header=T, sep="\t", check.names=F)

rt=as.matrix(rt)

rownames(rt)=rt[,1]

exp=rt[,2:ncol(rt)]

dimnames=list(rownames(exp),colnames(exp))

data=matrix(as.numeric(as.matrix(exp)),nrow=nrow(exp),dimnames=dimnames)

data=avereps(data)

#读取基因集文件

geneSets=getGmt(gmtFile, geneIdType=SymbolIdentifier())

#ssGSEA分析

ssgseaScore=gsva(data, geneSets, method='ssgsea', kcdf='Gaussian', abs.ranking=TRUE)

#对ssGSEA打分进行矫正

normalize=function(x){

return((x-min(x))/(max(x)-min(x)))}

ssgseaScore=normalize(ssgseaScore)

#输出ssGSEA打分结果

ssgseaOut=rbind(id=colnames(ssgseaScore), ssgseaScore)

write.table(ssgseaOut,file="ssGSEA.result.txt",sep="\t",quote=F,col.names=F)

#输出免疫热图

library(pheatmap)

inputFile="ssGSEA.result.txt" #ssGSEA的结果文件

setwd("~/liuxingchen/YMIPF/GEO/10ssGSEA/150910") #设置工作目录

#读取免疫细胞的打分文件

rt=read.table(inputFile, header=T, sep="\t", check.names=F, row.names=1)

#对样品分组

con=grepl("_con", colnames(rt), ignore.case=T)

treat=grepl("_treat", colnames(rt), ignore.case=T)

conData=rt[,con]

treatData=rt[,treat]

conNum=ncol(conData)

treatNum=ncol(treatData)

data=cbind(conData,treatData)

#绘制热图

Type=c(rep("Normal",conNum), rep("IPF",treatNum))

names(Type)=colnames(data)

Type=as.data.frame(Type)

pdf(file="heatmap.pdf", width=8, height=5)

pheatmap(data,

annotation=Type,

color=colorRampPalette(c(rep("blue",3), "white", rep("red",3)))(50),

cluster_cols=F,

show_colnames=F,

scale="row",

fontsize = 12,

fontsize_row=12,

fontsize_col=12)

dev.off()

#10免疫箱型图引用包

library(reshape2)

library(ggpubr)

library(limma)

library(GSEABase)

library(GSVA)

expFile="normalize.txt" #表达输入文件

gmtFile="immune.gmt" #免疫数据集文件

clusterFile="Cluster.txt" #m6A分型结果文件

setwd("~/liuxingchen/YMIPF/GEO/10ssGSEA") #设置工作目录

#读取表达输入文件,并对输入文件整理

rt=read.table(expFile, header=T, sep="\t", check.names=F)

rt=as.matrix(rt)

rownames(rt)=rt[,1]

exp=rt[,2:ncol(rt)]

dimnames=list(rownames(exp),colnames(exp))

data=matrix(as.numeric(as.matrix(exp)),nrow=nrow(exp),dimnames=dimnames)

data=avereps(data)

#读取基因集文件

geneSets=getGmt(gmtFile, geneIdType=SymbolIdentifier())

#ssGSEA分析

ssgseaScore=gsva(data, geneSets, method='ssgsea', kcdf='Gaussian', abs.ranking=TRUE)

#对ssGSEA打分进行矫正

normalize=function(x){

return((x-min(x))/(max(x)-min(x)))}

ssgseaScore=normalize(ssgseaScore)

#输出ssGSEA打分结果

ssgseaOut=rbind(id=colnames(ssgseaScore), ssgseaScore)

write.table(ssgseaOut,file="ssGSEA.result.txt",sep="\t",quote=F,col.names=F)

#读取分型的结果文件

cluster=read.table(clusterFile, header=T, sep="\t", check.names=F, row.names=1)

#数据合并

ssgseaScore=t(ssgseaScore)

sameSample=intersect(row.names(ssgseaScore), row.names(cluster))

ssgseaScore=ssgseaScore[sameSample,,drop=F]

cluster=cluster[sameSample,"Cluster",drop=F]

scoreCluster=cbind(ssgseaScore, cluster)

#把数据转换成ggplot2输入文件

data=melt(scoreCluster, id.vars=c("Cluster"))

colnames(data)=c("Cluster", "Immune", "Fraction")

#绘制箱线图

bioCol=c("#FF0000","#0066FF","#FF9900","#6E568C","#7CC767","#223D6C","#D20A13","#FFD121","#088247","#11AA4D")

bioCol=bioCol[1:length(levels(factor(data[,"Cluster"])))]

p=ggboxplot(data, x="Immune", y="Fraction", color="Cluster",

xlab="",

ylab="Immune infiltration",

legend.title="Type",

palette=bioCol)

p=p+rotate_x_text(50)

#输出图形文件

pdf(file="boxplot.pdf", width=10, height=5.5)

p+stat_compare_means(aes(group=Cluster),symnum.args=list(cutpoints = c(0, 0.001, 0.01, 0.05, 1), symbols = c("***", "**", "*", "")),label = "p.signif")

dev.off()

#11immuncor引用包

library(limma)

library(pheatmap)

library(reshape2)

library(ggpubr)

clusterFile="Cluster.txt" #m6A分型的结果文件

ssgseaFile="ssGSEA.result.txt" #ssGSEA的打分文件

setwd("~/liuxingchen/YMIPF/GEO/11immuncor") #设置工作目录

#读取分型的结果文件

cluster=read.table(clusterFile, header=T, sep="\t", check.names=F, row.names=1)

cluster=cluster[,-ncol(cluster)]

#读取ssGSEA的结果文件

ssgsea=read.table(ssgseaFile, header=T, sep="\t", check.names=F, row.names=1)

ssgsea=t(ssgsea)

#数据整理

sameSample=intersect(row.names(cluster), row.names(ssgsea))

cluster=cluster[sameSample,,drop=F]

ssgsea=ssgsea[sameSample,,drop=F]

#相关性检验

cor=cor(ssgsea, cluster, method="spearman")

#绘制相关性热图

pdf(file="heatmap.pdf", width=4, height=6)

pheatmap(cor,

color = colorRampPalette(c(rep("#6384E6",1), "white", rep("#D64D4D",1)))(100),

cluster_cols =F,

cluster_rows =F,

display_numbers = T,

show_colnames=T,

show_rownames=T,

angle_col =0,

fontsize=15,

fontsize_row=12,

fontsize_col=12)

dev.off()

#相关性分析

outTab=data.frame()

for(checkpiont in colnames(ssgsea)){

for(gene in colnames(cluster)){

x=as.numeric(ssgsea[,checkpiont])

y=as.numeric(cluster[,gene])

corT=cor.test(x,y,method="spearman")

cor=corT$estimate

pvalue=corT$p.value

text=ifelse(pvalue<0.001,"***",ifelse(pvalue<0.01,"**",ifelse(pvalue<0.05,"*","")))

outTab=rbind(outTab,cbind(Gene=gene, checkpiont=checkpiont, cor, text, pvalue))

}

}

#绘制相关性热图

outTab$Gene=factor(outTab$Gene, levels=colnames(cluster))

outTab$cor=as.numeric(outTab$cor)

pdf(file="checkpointCor.pdf", width=7, height=2.5)

ggplot(outTab, aes(checkpiont,Gene)) +

geom_tile(aes(fill = cor), colour = "grey", size = 1)+

scale_fill_gradient2(low = "#5C5DAF", mid = "white", high = "#EA2E2D") +

geom_text(aes(label=text),col ="black",size = 3) +

theme_minimal() + #去掉背景

theme(axis.title.x=element_blank(), axis.ticks.x=element_blank(), axis.title.y=element_blank(),

axis.text.x = element_text(angle = 90, hjust = 1, size = 9,face = "bold"), #x轴字体

axis.text.y = element_text(size = 9,face = "bold")) + #y轴字体

labs(fill =paste0("*** p<0.001","\n", "** p<0.01","\n", " * p<0.05","\n", "\n","Correlation")) + #设置图例

scale_x_discrete(position = "bottom") #X轴名称显示位置

dev.off()

#12免疫检查点

#引用包

library(limma)

library(reshape2)

library(ggplot2)

expFile="normalize.txt" #表达数据文件

geneFile="gene.txt" #免疫检查点基因列表文件

riskFile="Cluster.txt" #风险文件

setwd("~/liuxingchen/YMIPF/GEO/12ICB") #设置工作目录

#读取表达输入文件,并对输入文件整理

rt=read.table(expFile, header=T, sep="\t", check.names=F)

rt=as.matrix(rt)

rownames(rt)=rt[,1]

exp=rt[,2:ncol(rt)]

dimnames=list(rownames(exp), colnames(exp))

data=matrix(as.numeric(as.matrix(exp)), nrow=nrow(exp), dimnames=dimnames)

data=avereps(data)

#读取基因列表文件，获取免疫检查点相关基因的表达量

geneRT=read.table(geneFile, header=F, sep="\t", check.names=F)

sameGene=intersect(as.vector(geneRT[,1]), rownames(data))

data=t(data[sameGene,])

#读取风险文件

risk=read.table(riskFile, header=T, sep="\t", check.names=F, row.names=1)

sameSample=intersect(row.names(data), row.names(risk))

data=data[sameSample,,drop=F]

risk=risk[sameSample,3:(ncol(risk)-1),drop=F]

#相关性分析

outTab=data.frame()

for(checkpiont in colnames(data)){

for(gene in colnames(risk)){

x=as.numeric(data[,checkpiont])

y=as.numeric(risk[,gene])

corT=cor.test(x,y,method="spearman")

cor=corT$estimate

pvalue=corT$p.value

text=ifelse(pvalue<0.001,"***",ifelse(pvalue<0.01,"**",ifelse(pvalue<0.05,"*","")))

outTab=rbind(outTab,cbind(Gene=gene, checkpiont=checkpiont, cor, text, pvalue))

}

}

#绘制相关性热图

outTab$Gene=factor(outTab$Gene, levels=colnames(risk))

outTab$cor=as.numeric(outTab$cor)

pdf(file="checkpointCor.pdf", width=10.5, height=1.5)

ggplot(outTab, aes(checkpiont,Gene)) +

geom_tile(aes(fill = cor), colour = "grey", size = 1)+

scale_fill_gradient2(low = "#5C5DAF", mid = "white", high = "#EA2E2D") +

geom_text(aes(label=text),col ="black",size = 3) +

theme_minimal() + #去掉背景

theme(axis.title.x=element_blank(), axis.ticks.x=element_blank(), axis.title.y=element_blank(),

axis.text.x = element_text(angle = 90, hjust = 1, size = 9,face = "bold"), #x轴字体

axis.text.y = element_text(size = 9,face = "bold")) + #y轴字体

labs(fill =paste0("*** p<0.001","\n", "** p<0.01","\n", " * p<0.05","\n", "\n","Correlation")) + #设置图例

scale_x_discrete(position = "bottom") #X轴名称显示位置

dev.off()

#13免疫箱型图引用包

library(reshape2)

library(ggpubr)

library(limma)

library(GSEABase)

library(GSVA)

expFile="normalize.txt" #表达输入文件

gmtFile="immune.gmt" #免疫数据集文件

clusterFile="Cluster.txt" #m6A分型结果文件

setwd("~/liuxingchen/YMIPF/GEO/13immunfun") #设置工作目录

#读取表达输入文件,并对输入文件整理

rt=read.table(expFile, header=T, sep="\t", check.names=F)

rt=as.matrix(rt)

rownames(rt)=rt[,1]

exp=rt[,2:ncol(rt)]

dimnames=list(rownames(exp),colnames(exp))

data=matrix(as.numeric(as.matrix(exp)),nrow=nrow(exp),dimnames=dimnames)

data=avereps(data)

#读取基因集文件

geneSets=getGmt(gmtFile, geneIdType=SymbolIdentifier())

#ssGSEA分析

ssgseaScore=gsva(data, geneSets, method='ssgsea', kcdf='Gaussian', abs.ranking=TRUE)

#对ssGSEA打分进行矫正

normalize=function(x){

return((x-min(x))/(max(x)-min(x)))}

ssgseaScore=normalize(ssgseaScore)

#输出ssGSEA打分结果

ssgseaOut=rbind(id=colnames(ssgseaScore), ssgseaScore)

write.table(ssgseaOut,file="ssGSEA.result.txt",sep="\t",quote=F,col.names=F)

#读取分型的结果文件

cluster=read.table(clusterFile, header=T, sep="\t", check.names=F, row.names=1)

#数据合并

ssgseaScore=t(ssgseaScore)

sameSample=intersect(row.names(ssgseaScore), row.names(cluster))

ssgseaScore=ssgseaScore[sameSample,,drop=F]

cluster=cluster[sameSample,"Cluster",drop=F]

scoreCluster=cbind(ssgseaScore, cluster)

#把数据转换成ggplot2输入文件

data=melt(scoreCluster, id.vars=c("Cluster"))

colnames(data)=c("Cluster", "Immune", "Fraction")

#绘制箱线图

bioCol=c("#FF0000","#0066FF","#FF9900","#6E568C","#7CC767","#223D6C","#D20A13","#FFD121","#088247","#11AA4D")

bioCol=bioCol[1:length(levels(factor(data[,"Cluster"])))]

p=ggboxplot(data, x="Immune", y="Fraction", color="Cluster",

xlab="",

ylab="Immune infiltration",

legend.title="Type",

palette=bioCol)

p=p+rotate_x_text(50)

#输出图形文件

pdf(file="boxplot.pdf", width=10, height=5.5)

p+stat_compare_means(aes(group=Cluster),symnum.args=list(cutpoints = c(0, 0.001, 0.01, 0.05, 1), symbols = c("***", "**", "*", "")),label = "p.signif")

dev.off()

object.size("liuxingchen")

##################################################################################################################################

####################################### 数据导入 R.data ##########################################################################

## 进入工作目录 ##

setwd("~/liuxingchen/YMIPF/singlecell/GSE132771scsequencing/all")

getwd()

# 调用 Seurat包

library(Seurat)

library(tidyverse)

library(dplyr)

library(patchwork)

# 构建读取文件列表

####*************************** 需修改样本名 *******************************##

dir = c("normal1/", "normal2/","normal3/","IPF1/", "IPF2/","IPF3/") #

names(dir) = c("normal1", "normal2","normal3","IPF1", "IPF2","IPF3") #

####************************************************************************##

dir

# 循环读取

scRNA.list <- list()

for(i in 1:length(dir)){

# Read10X函数加载 10X平台数据

scRNA.counts <- Read10X(data.dir = dir[i])

# 使用 CreateSeuratObject 函数创建 多个 Seurat 对象

scRNA.list[[i]] <- CreateSeuratObject(scRNA.counts, min.cells = 3, min.features =200)

rm(scRNA.counts)

# scRNA.counts要求没有 normalized project="XXX" #项目名字

# *** min.cells 过滤：每个基因至少要在3个细胞中检测到

# *** min.features 过滤：每个细胞至少要检测到200个基因

}

#查看细胞总数：

for(i in 1:length(scRNA.list)) {

print(table(scRNA.list[[i]]@meta.data$orig.ident))

}

head(scRNA.list[[1]][[]]) # 即 scRNA.list[[]] 层级结构中 meta.data 中内容

# nCount_RNA 所有基因的表达量之和（代表该细胞的测序深度）

# nFeature_RNA 有多少基因表达（每个细胞中能检测到的基因）

setwd("~/liuxingchen/YMIPF/singlecell/GSE132771scsequencing/RPCA-SCT")

save(scRNA.list,file='scRNA.list-multi.Rdata')

rm(scRNA.list,dir,i)

##################################################################################################################################

##################################################################################################################################

######################################### 细胞过滤 ###############################################################################

##（去除线粒体基因较多的细胞[可能走向衰老]、血红细胞[成熟红细胞没有细胞核]）

load('scRNA.list-multi.Rdata')

head(scRNA.list[[1]][[]])

################################################################################

# 计算比例

for (i in 1:length(scRNA.list)) {

# 计算细胞中线粒体比例，将

scRNA.list[[i]][["percent.mt"]] <- PercentageFeatureSet(scRNA.list[[i]], pattern = "^MT-")

# 若为人，则改为 "^MT-" # 若为小鼠，则改为 "^mt-"

# "^MT-" 正则表达，匹配基因名 “MT-” 开头的基因（线粒体基因）

# 在 meta.data 中增加 percent.mt 这一列

# 计算红细胞比例

HB_m <- match(c("HBA1","HBA2","HBB","HBD","HBE1","HBG1","HBG2","HBM","HBQ1","HBZ"), rownames(scRNA.list[[i]]@assays$RNA))

HB.genes <- rownames(scRNA.list[[i]]@assays$RNA)[HB_m]

HB.genes <- HB.genes[!is.na(HB.genes)]

rm(HB_m)

#搜索常见的 血红细胞基因

scRNA.list[[i]][["percent.HB"]]<-PercentageFeatureSet(scRNA.list[[i]], features=HB.genes)

rm(HB.genes)

# 计算比例，并添加到 meta.data 中增加 percent.HB 一列

}

head(scRNA.list[[1]][[]])

# 可视化 nFeature_RNA、nCount_RNA、线粒体基因比例、血红细胞基因比列

nC_nF_MT_HB_plot.list = list()

for (i in 1:length(scRNA.list)) {

col.num <- length(levels(scRNA.list[[i]]@active.ident))

nC_nF_MT_HB_plot = VlnPlot(scRNA.list[[i]],

features = c( "nCount_RNA", "nFeature_RNA", "percent.mt","percent.HB"),

cols =rainbow(col.num), pt.size = 0.01, ncol = 4) +

theme(axis.title.x=element_blank(),

axis.text.x=element_blank(),

axis.ticks.x=element_blank())

nC_nF_MT_HB_plot.list[[i]] = nC_nF_MT_HB_plot

rm(col.num,nC_nF_MT_HB_plot)

}

CombinePlots(plots = nC_nF_MT_HB_plot.list, nrow=length(scRNA.list), legend="none")

rm(nC_nF_MT_HB_plot.list)

# 查看相关性 nFeature_RNA、nCount_RNA、线粒体基因比例、血红细胞基因比列间相关性

nC_nF_MT_HB_correlation.list = list()

for (i in 1:length(scRNA.list)) {

plot1=FeatureScatter(scRNA.list[[i]], feature1 = "nCount_RNA", feature2 = "nFeature_RNA")

plot2=FeatureScatter(scRNA.list[[i]], feature1 = "nCount_RNA", feature2 = "percent.mt")

plot3=FeatureScatter(scRNA.list[[i]], feature1 = "nCount_RNA", feature2 = "percent.HB")

nC_nF_MT_HB_correlation = CombinePlots(plots = list(plot1, plot2, plot3), nrow=1, legend="none")

nC_nF_MT_HB_correlation.list[[i]] = nC_nF_MT_HB_correlation

rm(plot1,plot2,plot3, nC_nF_MT_HB_correlation)

}

CombinePlots(plots = nC_nF_MT_HB_correlation.list, nrow=length(scRNA.list), legend="none")

rm(nC_nF_MT_HB_correlation.list)

################################################################################

# 根据可视化结果 结合实际情况，选择过滤阈值

for (i in 1:length(scRNA.list)) {

scRNA.list[[i]] <- subset(scRNA.list[[i]],

subset = nFeature_RNA > 500 &

percent.mt < 20 &

percent.HB < 3)

}

#可再次运行可视化代码，查看质控前后对比。默认参数nFeature_RNA > 500 & nCount_RNA > 1000 &percent.mt < 20 & percent.HB < 3

save(scRNA.list,file='after_quality_control-mulit.Rdata')

rm(scRNA.list,i)

setwd("../")

##################################################################################################################################

##################################################################################################################################

############################ 归一化，挑选高变基因整合不同样本，消除批次效应，中心化 ##############################################

## 进入工作目录 ##

setwd("~/liuxingchen/YMIPF/singlecell/GSE132771scsequencing/RPCA-SCT")

getwd()

# 调用R包

library(Seurat)

library(tidyverse)

library(dplyr)

library(patchwork)

library(glmGamPoi)

################################################################################

##多线程设置##

library(future)

plan("multiprocess", workers =4)

options(future.globals.maxSize = 2000 * 4096^2)

################################################################################

load('after_quality_control-mulit.Rdata')

head(scRNA.list[[1]][[]])

##################################################################################################################################

################################################################################

#安装 glmGamPoi

# chooseBioCmirror()

# BiocManager::install("glmGamPoi")

################################################################################

# 使用 SCTransform # 归一化、寻找高变基因、中心化数据

scRNA.list <- lapply(X = scRNA.list, FUN = SCTransform, method = "glmGamPoi")

features <- SelectIntegrationFeatures(object.list = scRNA.list, nfeatures = 3000)

scRNA.list <- PrepSCTIntegration(object.list = scRNA.list, anchor.features = features)

scRNA.list <- lapply(X = scRNA.list, FUN = RunPCA, features = features)

################################################################################

# 寻找锚定点

scRNA.anchors <- FindIntegrationAnchors(object.list = scRNA.list,

normalization.method = "SCT",

anchor.features = features,

dims = 1:30, reduction = "rpca",

k.anchor = 20)

# k.anchor 参数为整合强度（默认为5 可以设置到20）

# 整合数据

scRNA <- IntegrateData(anchorset = scRNA.anchors,normalization.method = "SCT", dims = 1:30)

# 查看整合后的数据

scRNA.table = scRNA[[]]

rm(scRNA.table, scRNA.anchors, features,scRNA.list)

################################################################################

# PCA降维 #

scRNA <- RunPCA(scRNA, verbose = T)

scRNA.table = scRNA[[]]

save(scRNA,file='scRNA-Normalize-ScaleData.Rdata')

#################################################################################################################################

## 可视化，看整合是否符合标准，查看不同批次样本是否发生聚类

###**************************************************************************###

# PCA 可视化 ###

DimPlot(scRNA, reduction = "pca", group.by="orig.ident") ###

###

# UMAP 可视化 ###

scRNA <- RunUMAP(scRNA, dims = 1:20 ) ###

DimPlot(scRNA, reduction = "umap", group.by = "orig.ident") ###

###**************************************************************************###

rm(scRNA,scRNA.table)

##################################################################################################################################

###################################### 去除细胞周期的影响 ########################################################################

## 进入工作目录 ##

setwd("~/liuxingchen/BRCA1/scsequencing/E220725008")

getwd()

# 调用 R包

library(Seurat)

library(tidyverse)

library(dplyr)

library(patchwork)

################################################################################

##多线程设置##

library(future)

plan("multiprocess", workers =4)

options(future.globals.maxSize = 2000 * 4096^2)

################################################################################

####*************************************************************************###

# 单个样本 or 锚定整合样本 导入 #

load('scRNA-Normalize-ScaleData.Rdata') #

####******************************** or *********************************###

# # Harmony 整合结果导入 #

load('scRNA-Normalize-ScaleData-Harmony.Rdata') #

scRNA <- scRNA_harmony #

rm(scRNA_harmony) #

####*************************************************************************###

# 查看数据

scRNA.table = scRNA[[]]

################################################################################

# G2期、M期基因 与 高变基因 取交集

g2m_genes = CaseMatch(search = cc.genes$g2m.genes, match = rownames(scRNA))

# cc.genes 为 Seurat 自带的周期函数列表

# S期基因 与 高变基因 取交集

s_genes = CaseMatch(search = cc.genes$s.genes, match = rownames(scRNA))

# 对交集中的基因进行打分 并 可视化结果

scRNA <- CellCycleScoring(object=scRNA, g2m.features=g2m_genes, s.features=s_genes)

VlnPlot(scRNA, features = c("G2M.Score","S.Score"), ncol = 2)

# 只对周期基因进行PCA降维 并 可视化结果

scRNA.cc.genes <- RunPCA(scRNA, features = c(s_genes, g2m_genes))

DimPlot(scRNA.cc.genes, reduction = "pca", group.by = "Phase")

# 观察可视化结果，是否符合要求（ G2 M S 期细胞之间是否发生聚类，若打多数聚为一团，则符合）

# 若不符合要求，需要将 "S.Score", "G2M.Score" 写入 scRNA，后期筛选

################################################################################

# 去除周期基因的影响

scRNA <- ScaleData(scRNA, vars.to.regress = c("S.Score", "G2M.Score"), features = rownames(scRNA))

rm(g2m_genes, s_genes, scRNA.cc.genes)

# 可再次可视化，观察是否消除影响

save(scRNA,file='scRNA_after_cc.genes.Rdata')

rm(scRNA, scRNA.table)

##################################################################################################################################

##################################################################################################################################

################################## 聚类分析 Neighbors & Clusters $ 寻找 Marker基因 ############################################

##################################################################################################################################

## 进入工作目录 ##

setwd("~/liuxingchen/BRCA1/scsequencing/E220725008")

getwd()

# 调用 Seurat包

library(Seurat)

library(tidyverse)

library(dplyr)

library(patchwork)

################################################################################

##多线程设置##

library(future)

plan("multiprocess", workers =4)

options(future.globals.maxSize = 2000 * 4096^2)

################################################################################

load('scRNA_after_cc.genes.Rdata')

scRNA.table = scRNA[[]]

################################################################################

####*************************************************************************###

# 选择合适的主成分数 #

ElbowPlot(scRNA, ndims=50, reduction="pca") #

pc.num=1:20 #

scRNA <- FindNeighbors(scRNA, reduction = "pca", dims = pc.num) %>% #

FindClusters(resolution = 0.4) #

#

####******************************** or *********************************###

# # Harmony 整合结果导入 #

# # 选择适合的主成分数 #

ElbowPlot(scRNA, ndims=50, reduction="harmony") #

pc.num=1:20 #

scRNA <- FindNeighbors(scRNA, reduction = "harmony", dims = pc.num) %>% #

FindClusters(resolution = 0.5) #

#******************************************************************************#

scRNA.table = scRNA[[]] #

#################################################################################################################################

# 聚类（两种方法） ##

# pc.num = 1:30

#方法一：UMAP：统一流形逼近与投影

####*********************************************************************###

scRNA <- RunUMAP(scRNA,reduction = "pca", dims = pc.num) #

####***************************** or ********************************###

# # Harmony 整合结果导入 #

scRNA <- RunUMAP(scRNA, reduction = "harmony", dims = pc.num) #

#**************************************************************************#

#保存 UMAP 聚类结果数据

embed_umap <- Embeddings(scRNA, 'umap')

write.csv(embed_umap,'embed_umap.csv')

#查看不同细胞类型聚类情况

p_umap = DimPlot(scRNA, reduction = "umap")

DimPlot(scRNA, reduction = "umap",label = TRUE)

#保存图片

ggsave("UMAP.png", plot = p_umap, width = 8, height = 7)

#方法二：tsne：t-分布式随机邻居嵌入

#**************************************************************************#

scRNA = RunTSNE(scRNA, dims = pc.num) #

####***************************** or ********************************###

# # Harmony 整合结果导入 #

scRNA <- RunTSNE(scRNA, reduction = "harmony", dims = pc.num) #

#**************************************************************************#

# 保存 tsne 聚类结果数据

embed_tsne <- Embeddings(scRNA, 'tsne')

write.csv(embed_tsne,'embed_tsne.csv')

#查看不同细胞类型聚类情况

p_tsne = DimPlot(scRNA, reduction = "tsne")

DimPlot(scRNA, reduction = "tsne",label = TRUE)

# 保存图片

# ggsave("tSNE.png", plot = p_tsne, width = 8, height = 7)

#查看系统发育树

PlotClusterTree(BuildClusterTree(scRNA))

rm(embed_tsne,embed_umap,p_tsne,p_umap,pc.num)

###***************************** 可选步骤 ***********************************###

########## 合并样本中多重复组 #################################################

# 查看所有样本

table(scRNA.table$orig.ident)

# 合并数据 添加 Sample.type列

scRNA@meta.data[which(scRNA@meta.data$orig.ident=="IPF1"),"Sample.type"] <- "IPF"

scRNA@meta.data[which(scRNA@meta.data$orig.ident=="IPF2"),"Sample.type"] <- "IPF"

scRNA@meta.data[which(scRNA@meta.data$orig.ident=="IPF3"),"Sample.type"] <- "IPF"

scRNA@meta.data[which(scRNA@meta.data$orig.ident=="normal1"),"Sample.type"] <- "Normal"

scRNA@meta.data[which(scRNA@meta.data$orig.ident=="normal2"),"Sample.type"] <- "Normal"

scRNA@meta.data[which(scRNA@meta.data$orig.ident=="normal3"),"Sample.type"] <- "Normal"

# 查看合并后样本

scRNA.table = scRNA[[]]

##***************************************************************************###

save(scRNA,file='scRNA_Clusters.Rdata')

rm(scRNA.table)

#################################################################################

##################################################################################################################################

################################### SingleR 细胞注释 ############################################################################

##################################################################################################################################

## 进入工作目录 ##

setwd("~/liuxingchen/BRCA1/scsequencing/E220725008/传统锚定点")

getwd()

# 调用 Seurat包

library(Seurat)

library(tidyverse)

library(dplyr)

library(patchwork)

################################################################################

##多线程设置##

library(future)

plan("multiprocess", workers =4)

options(future.globals.maxSize = 2000 * 4096^2)

################################################################################

load('scRNA_Clusters.Rdata')

scRNA.table = scRNA[[]]

################################################################################

# 安装 SingleR 包

# chooseBioCmirror()

# BiocManager::install("SingleR")

library(SingleR)

#################################################################################################################################

## 使用 SingleR 数据库 细胞类型的注释文件 ##

#################################################################################################################################

###SingleR 数据库内置数据集：

#cg=celldex::BlueprintEncodeData() # 人

# cg=DatabaseImmuneCellExpressionData() # 人

# cg=NovershternHematopoieticData() # 人

# cg=MonacoImmuneData() # 人

cg=celldex::ImmGenData() # 小鼠

# cg=MouseRNAseqData() # 小鼠

# cg=HumanPrimaryCellAtlasData() # 人

# cg<-celldex::HumanPrimaryCellAtlasData()#选取我们要使用的参考数据集

refdata<-cg

??celldex

# 导入 SingleR 人类数据集

# load("ref_Human_all.RData")

# refdata <- ref_Human_all

# rm(ref_Human_all)

# 提取 RNA 的转录表达数据

testdata <- GetAssayData(scRNA, slot="data")

# 提取scRNA数据中的seurat_clusters

clusters <- scRNA@meta.data$seurat_clusters

# 开始用 SingleR 分析

cellpred <- SingleR(test = testdata, ref = refdata,

labels = refdata$label.main,

method = "cluster", clusters = clusters,

# method 选择 "cluster"，一个 cluster 对应一种细胞类型

assay.type.test = "logcounts", assay.type.ref = "logcounts")

# 提取 SingleR 分析结果，注释制作细胞类型的注释文件

celltype = data.frame(ClusterID=rownames(cellpred), celltype=cellpred$labels, stringsAsFactors = FALSE)

celltype

rm(cellpred,refdata,testdata,clusters)

#保存注释信息

write.csv(celltype,"HumanPrimaryCell-singleR.csv",row.names = FALSE)

################################################################################

###############################################################################

# 把singler的注释写到 meta.data中

scRNA@meta.data$cell.type ="NA"

for(i in 1:nrow(celltype)){

scRNA@meta.data[which(scRNA@meta.data$seurat_clusters == celltype$ClusterID[i]),'cell.type'] <- celltype$celltype[i]

}

rm(celltype,i)

scRNA.table = scRNA[[]]

#可视化结果

P_umap <- DimPlot(scRNA, group.by="cell.type",reduction = "umap", label=T, label.size=5)

P_umap

# ggsave("output/UMAP-SingleR-cell_type.png", P_umap, width=10 ,height=8)

P_tsne <- DimPlot(scRNA, group.by="cell.type",reduction = "tsne", label=T, label.size=5)

P_tsne

# ggsave("output/TSNE-SingleR-cell_type.png", P_umap, width=10 ,height=8)

save(scRNA,file='scRNA_SingleR_annotation.Rdata')

rm(P_tsne,P_umap,scRNA.table,scRNA)

#################################################################################################################################

FeaturePlot(scRNA,features = c('CFH'))

VlnPlot(scRNA, features = c('CFH','FHL2'), slot = "counts", log = TRUE)

VlnPlot(scRNA,features = c('CFH','FHL2'))

VlnPlot(scRNA,features = c('PER3','RORA','RORB','RORC','TIMELESS'))

#################################################################################################################################

## 使用 SingleR 自定义 细胞类型的注释文件 ##

#################################################################################################################################

###加载所需要的包

library(Seurat)

library(tidyverse)

library(dplyr)

library(patchwork)

library(SingleR)

# BiocManager::install("org.Hs.eg.db")

library(org.Hs.eg.db)

# BiocManager::install("scRNAseq")

library(scRNAseq)

################################################################################

# 这里，我们使用来自 scRNAseq 包中的两个人类胰腺数据集。

# 目的是使用一个预先标记好的数据集对另一个未标记的数据集进行细胞类型注释。

# 准备参考数据集

# ———— 使用Muraro et al.(2016)的数据作为我们的参考数据集。

sceM <- MuraroPancreasData()

# 注意此处为另一种单细胞数据的S4对象

# 移除未标记的细胞

sceM <- sceM[,!is.na(sceM$label)]

# 对数据进行简单的归一化处理，使用 scater包

# BiocManager::install("scater")

# BiocManager::install("scran")

library(scater)

library(scran)

sceM <- logNormCounts(sceM)

# 准备测试数据集

# ————— 使用Grun et al.(2016)的数据作为测试数据集。

sceG <- GrunPancreasData()

# 去除表达量为 0 的细胞

sceG <- sceG[,colSums(counts(sceG)) > 0]

# 对数据进行简单的归一化处理

sceG <- logNormCounts(sceG)

#为了加快分析的速度，我们挑选前200个细胞进行分析。

sceG <- sceG[,1:500]

# 使用SingleR函数进行细胞类型注释，并指定de.method="wilcox"检测方法

pred.grun <- SingleR(test=sceG, ref=sceM, labels=sceM$label, de.method="wilcox")

# 查看细胞类型注释的预测结果

table(pred.grun$labels)

plotScoreHeatmap(pred.grun)

#使用pruneScores()函数删除那些质量低下或模棱两可的分配

remove.cell <- pruneScores(pred.grun)

summary(remove.cell)

#低于3个中位数绝对偏差（MAD）值来定义适当的阈值

plotScoreDistribution(pred.grun, ncol =5, show.nmads = 3)

##如果必须调整某些参数，我们可以直接使用调整后的参数来调用pruneScores()函数。

#这里，我们将要丢弃的标签设置为NA，这也是SingleR()函数如何在pruned.labels中标记此类标签的方式。

new.pruned <- pred.grun$labels

new.pruned[pruneScores(pred.grun, nmads=5)] <- NA

table(new.pruned, useNA="always")

all.markers <- metadata(pred.grun)$de.genes

sceG$labels <- pred.grun$labels

# Beta cell-related markers

plotHeatmap(sceG, order_columns_by="labels",

features=unique(unlist(all.markers$beta)))

##展示各个cluster的marker

for (lab in unique(pred.grun$labels)) {

plotHeatmap(sceG, order_columns_by=list(I(pred.grun$labels)),

features=unique(unlist(all.markers[[lab]])))

}

#################################################################################################################################

#######################################################################################################################

# 进入工作目录

setwd("~/liuxingchen/YMIPF/singlecell/GSE132771scsequencing/RPCA-SCT")

getwd()

# 加载所需要的包

library(Seurat)

library(tidyverse)

library(dplyr)

library(patchwork)

library(RColorBrewer)

library(reshape2)

# 加载数据

load("scRNA_Manual_annotation.Rdata")

scRNA<-scRNA.ANN

rm(scRNA.ANN)

sc.table <- scRNA[[]]

################################################################################

############ 不同 样本 or 样本分组 中 细胞类型 所占比例 绘图 #############

################################################################################

####********************************************************************************###

# 对数据中 不同批次样本类型（Sample.tpye） 与 样本中细胞类型（celltype）进行提取 #

pB2_df <- table(scRNA@meta.data$cell.type,scRNA@meta.data$Sample.type) %>% melt() #

table(pB2_df$Var2) #

# 对样本类型进行排序 #

sample = c("IPF","Normal") #

####*********************************** or *************************************###

# # 对数据中 样本分组类型（type） 与 样本中细胞类型（celltype）进行提取 #

pB2_df <- table(scRNA@meta.data$cell.type,scRNA@meta.data$orig.ident) %>% melt() #

table(pB2_df$Var2) #

# # 对样本类型进行排序 #

sample = c("IPF1","IPF2","IPF3","normal1","normal2","normal3") #

####********************************************************************************###

# 对列名进行更改

colnames(pB2_df) <- c("Cluster","Sample","Number")

# 将 Sample 即 样本类型 设置为因子

pB2_df$Sample <- factor(pB2_df$Sample,levels = sample)

rm(sample)

# 将 Cluster 即 细胞类型 设置为因子

# 对细胞类型进行排序

table(pB2_df$Cluster)

cluster = c("Fibroblasts","Smooth_muscle_cells","Epithelial_cells","Endothelial_cells","Monocyte","Macrophage",

"Neutrophils","T_cells","B_cell",

"NK_cell")

# 转换为因子

pB2_df$Cluster <- factor(pB2_df$Cluster,levels = cluster)

rm(cluster)

# 去除数据中的空值

pB2_df=na.omit(pB2_df)

# 绘制不同样本分组中类型中各细胞群所占比例

pB2 <- ggplot(data = pB2_df, aes(x = Number, y = Sample, fill = Cluster)) +

# fill 指定按什么分组

geom_bar(stat = "identity", width=0.8,position="fill") +

# 绘制柱状图

ggsci::scale_fill_simpsons() +

# 使用 ggsci 的颜色 进行柱状图填充

theme_bw()+

theme(panel.grid =element_blank()) +

# 去除背景网格

labs(x="Ratio",y="")+

# 修改 X Y轴 标签

theme(axis.text.y = element_text(size=15, colour = "black"))+

# 修改 y轴字体

theme(axis.text.x = element_text(size=15, colour = "black"))+

# 修改 y轴字体

theme(axis.text.x.bottom = element_text(hjust =0.5, vjust = 0.5, angle = 0))

# 修改 x轴 字体 角度与位置

pB2

# 绘制不同细胞类型 中 样本所占比例

pB2 <- ggplot(data = pB2_df, aes(x = Cluster, y = Number, fill = Sample)) +

# fill 指定按什么分组

geom_bar(stat = "identity", width=0.8,position="fill") +

# 绘制柱状图

ggsci::scale_fill_simpsons() +

# 使用 ggsci 的颜色 进行柱状图填充

theme_bw()+

theme(panel.grid =element_blank()) +

# 去除背景网格

labs(x="",y="Ratio")+

# 修改 X Y轴 标签

theme(axis.text.y = element_text(size=12, colour = "black"))+

# 修改 y轴字体

theme(axis.text.x = element_text(size=12, colour = "black"))+

# 修改 y轴字体

theme(axis.text.x.bottom = element_text(hjust = 1, vjust = 1, angle = 45))

# 修改 x轴 字体 角度与位置

pB2

rm(pB2_df, pB2)

################################################################################

################################################################################

######## 不同分组 中 不同细胞数量所占比列 及 标准差 绘制 #############

################################################################################

## 定义 top bottom 函数

top<-function(x){return(mean(x)+sd(x)/sqrt(length(x)))}

bottom<-function(x){return(mean(x)-sd(x)/sqrt(length(x)))}

# 查看细胞数量

cell_num <- table(scRNA@meta.data$cell.type,scRNA@meta.data$orig.ident)

cell_num

# 计算每种类型细胞 所占样本的百分比

cell_percent = t(t(cell_num)/rowSums(t(cell_num)))

cell_percent

# 转化为数据框

cell_data = as.data.frame(as.table(t(cell_percent)))

colnames(cell_data) = c("sample","celltype","Freq")

rm(cell_num,cell_percent)

"IPF1","IPF2","IPF3","normal1","normal2","normal3"

# 添加分组

cell_data[which(cell_data$sample=="IPF1"),"group"] <- "IPF"

cell_data[which(cell_data$sample=="IPF2"),"group"] <- "IPF"

cell_data[which(cell_data$sample=="IPF3"),"group"] <- "IPF"

cell_data[which(cell_data$sample=="normal1"),"group"] <- "Normal"

cell_data[which(cell_data$sample=="normal1"),"group"] <- "Normal"

cell_data[which(cell_data$sample=="normal1"),"group"] <- "Normal"

Sample.list = c("IPF","Normal")

# 查看分组情况

table(cell_data$group)

# 去除NA值

cell_data=na.omit(cell_data)

Cell_type_num_plot.list = list()

for (i in 1:length(Sample.list)) {

# 按分组提取数据

dose<-cell_data[which(cell_data$group==Sample.list[i]),]

# 绘制柱状图

Cell_type_num_plot <- ggplot(data=dose,aes(x=celltype,y=Freq,fill=celltype))+

stat_summary(geom = "bar",fun = "mean",position = position_dodge(0.9))+

stat_summary(geom = "errorbar",

fun.min = bottom,

fun.max = top,

position = position_dodge(0.9),

width=0.2)+

scale_y_continuous(expand = expansion(mult = c(0,0.1)))+

theme_bw()+

theme(panel.grid = element_blank())+

labs(x="Cell type",y="Proportion")+

geom_point(data=dose,aes(celltype,Freq),size=3,pch=19)+

theme(axis.text.x.bottom = element_text(hjust = 1, vjust = 1, angle = 45))+

ggtitle(Sample.list[i])+

ggsci::scale_fill_simpsons()

Cell_type_num_plot.list[[i]] = Cell_type_num_plot

rm(Cell_type_num_plot)

}

CombinePlots(plots = Cell_type_num_plot.list, nrow=length(Sample.list))

rm(i,Sample.list,dose,Cell_type_num_plot.list)

rm(cell_data,scRNA,sc.table)

rm(bottom,top)

################################################################################

#######################################################################################################################

# 进入工作目录

setwd("~/liuxingchen/YFgastricCA/P1P2vsP5P6")

getwd()

################################################################################

#加载所需要的包

library(Seurat)

library(tidyverse)

library(dplyr)

library(patchwork)

library(cowplot)

library(reshape2)

library(RColorBrewer)

#######################################################################################################################

# 导入数据

load('scRNA_Manual_annotation.Rdata')

scRNA<-scRNA.ANN

rm(scRNA.ANN)

# 查看数据

scRNA.table = scRNA[[]]

#######################################################################################################################

############ 绘制 不同 细胞类型 基因表达差异 峰峦图 & 小提琴图 ####################################################

###************************************************##

# 将 细胞注释类型 转换为 ident #

Idents(scRNA)=scRNA@meta.data$cell.type #

###******************* or **********************##

# # 将 细胞聚类类型 转换为 ident #

# Idents(scRNA)=scRNA@meta.data$seurat_clusters #

###************************************************##

# 需要查看的基因

#marker基因

gene.list = c("CFH","FHL2")

gene.list = c("ACTA2","COL1A1")

# 查看现有横坐标顺序

table(Idents(scRNA))

# 自定义顺序

My_levels <- c("Fibroblasts","Smooth_muscle_cells","Epithelial_cells",

"Endothelial_cells","Monocyte","Macrophage","Neutrophils",

"T_cells","B_cell","NK_cell")

# Seurat自带小提包绘图 #########################################################

Idents(scRNA) <- factor(Idents(scRNA), levels= My_levels)

DefaultAssay(scRNA) <- "RNA" # 修改默认矩阵为原始矩阵

VlnPlot(scRNA, features = gene.list, pt.size = 0, ncol = 1,log=T )&

scale_x_discrete("")

#theme(axis.text.x.bottom = element_blank())

VlnPlot(scRNA, features = c("ACTA2"),pt.size = 0, ncol = 1)&

scale_x_discrete("")

# 查看 不同分组中 各个细胞类型中 基因表达情况 对比

library(ggpubr)

VlnPlot(scRNA,features = gene.list,

split.by = "Sample.type",

pt.size = 0,

ncol = 1,log=T)&

stat_compare_means(method = "t.test",label = "p.format",

label.x.npc ="center",size = 4)&

#stat_boxplot()&

theme(legend.position = "right")

# 查看 不同分组中 各个细胞类型中 特定基因表达情况 对比带P值

# VlnPlot(object = scRNA,features = c("HSD17B11"),

# slot = "data",group.by = "cell.type",

# split.by = "Sample.type",

# pt.size = 0,

# ncol = 1)+

# stat_compare_means(method = "t.test", label = "p.format",

# label.x.npc ="center",size = 2.5)+

# stat_boxplot()

# 自定义绘制小提琴图 #########################################################

# 提取需要绘制的基因

table(Idents(scRNA))

vln.df=as.data.frame(scRNA[["RNA"]]@data[gene.list,])

# 将基因名添加到数据最后一列

vln.df$gene=rownames(vln.df)

# 宽窄 数据框 转换

vln.df=melt(vln.df,id="gene")

# 修改列名

colnames(vln.df)[c(2,3)]=c("barcode","exp")

# 提取 meta.data

anno=scRNA@meta.data

# 添加 barcode列

anno$barcode=rownames(anno)

# 将 vln.df 和 meta.data 进行合并

vln.df=inner_join(vln.df,anno,by="barcode")

# 将基因转换为因子，为了控制画图的基因顺序

vln.df$gene=factor(vln.df$gene,levels = gene.list)

# 将细胞类型转换为因子，为了控制画图的基因顺序

vln.df$cell.type=factor(vln.df$cell.type,levels = My_levels)

## 绘制图像颜色较少

vln.df%>%

ggplot(aes(cell.type,exp))+geom_violin(aes(fill=gene),scale = "width")+

facet_grid(vln.df$gene~.,scales = "free_y")+

scale_fill_brewer(palette = "Set3",direction = 1)+

scale_x_discrete("")+scale_y_continuous("")+

theme_bw()+

theme(axis.text.x.bottom = element_text(angle = 45,hjust = 1,vjust = 1),

panel.grid.major = element_blank(),

panel.grid.minor = element_blank(),

legend.position = "none")

## 绘制图像-增加更多颜色

colourcount<-length(gene.list)

vln.df%>%

ggplot(aes(cell.type,exp))+geom_violin(aes(fill=gene),scale = "width")+

facet_grid(vln.df$gene~.,scales = "free_y")+

scale_fill_manual(values=colorRampPalette(brewer.pal(8,"Set2"))(colourcount))+

scale_x_discrete("")+scale_y_continuous("")+

theme_bw()+

theme(axis.text.x.bottom = element_text(angle = 45,hjust = 1,vjust = 1),

panel.grid.major = element_blank(),

panel.grid.minor = element_blank(),

)

## 将每个基因的表达的量用颜色表示

vln.df$cell.type.gene=paste(vln.df$cell.type,vln.df$gene,sep = "_")

stat.df=as.data.frame(vln.df%>%

dplyr::group_by(cell.type,gene)%>%

dplyr::summarize(mean=mean(exp)))

colnames(stat.df)

stat.df$cell.type.gene=paste(stat.df$cell.type,stat.df$gene,sep = "_")

stat.df=stat.df[,c("mean","cell.type.gene")]

vln.df=inner_join(vln.df,stat.df,by="cell.type.gene")

vln.df$mean=ifelse(vln.df$mean > 3, 3, vln.df$mean)

vln.df%>%

ggplot(aes(cell.type,exp))+geom_violin(aes(fill=mean),scale = "width")+

facet_grid(vln.df$gene~.,scales = "free_y")+

scale_fill_gradient(limits=c(0,3),low = "darkblue",high = "red3")+

scale_x_discrete("")+scale_y_continuous("",expand = c(0.02,0))+

theme_bw()+

theme(axis.text.x.bottom = element_text(angle = 45,hjust = 1,vjust = 1),

panel.grid.major = element_blank(),

panel.grid.minor = element_blank())

rm(vln.df,stat.df,anno)

# 绘制峰峦图 ###################################################################

RidgePlot(scRNA, features = gene.list, ncol = 2)

rm(gene.list,My_levels)

rm(scRNA,scRNA.table)

#######################################################################################################################
